# Green hybrid polymeric magnetic nanocomposite from natural polycationic polysaccharides for sustainable alum sludge conditioning

**DOI:** 10.1038/s41598-026-35765-2

**Published:** 2026-02-02

**Authors:** Maha A. Tony, Zahraa A. Elsayed, Hamed M. Abdel-Bary, Jiayu Xin, Xingmei Lu, Mai K. Fouad, Ibrahim E.T. El-Sayed

**Affiliations:** 1 Advanced Materials/Solar Energy and Environmental Sustainability (AMSEES) Laboratory, Basic Engineering Science Department, Faculty of Engineering, Menoufia University, Shebin El-Kom, Egypt; 2 Planning & Construction of Smart Cities Program, Faculty of Engineering, Menoufia National University, Menoufia, 32651 Egypt; 3 Chemistry department, Faculty of Science, Menoufia University, Shebin El-Kom, Egypt; 4 Beijing Key Laboratory of Solid-State Battery and Energy Storage Process, CAS Key Laboratory of Green Process and Engineering, Institute of Process Engineering, Chinese Academy of Sciences, Beijing, 100190 China; 5 School of Chemical Engineering, University of Chinese Academy of Sciences, Beijing, 100049 China; 6 School of Chemistry and Chemical Engineering, University of Chinese Academy of Sciences, Beijing, 100049 China; 7 Chemical Engineering department, Faculty of Engineering, Cairo University, Giza, Egypt

**Keywords:** Aluminum based residue, Chitosan based fenton, Oxidation, Dewatering, Advanced oxidation processes, Chemistry, Engineering, Environmental sciences, Materials science

## Abstract

The disposal of aluminum nature-based sludge that is so called alum sludge (AS) from water-works treatment plants for water drinking purposes is one of the expensive sectors in treatment plant due to the high-water content. Thus, dewatering is essential for sludge volume reduction, which requires further treatment and drying costs prior to sludge disposal. Based on the criteria of advanced oxidation processes (AOP), alum sludge is subjected to polycationic polysaccharide-magnetic catalyst as a catalyst-based Fenton oxidation treatment and its effect and mechanism on sludge dewatering were assessed in the current work. Polycationic polysaccharide, chitosan augmented with magnetite in various proportions named CSP@Fe_3_O_4_(1–1), CSP@Fe_3_O_4_-(2 − 1) and CSP@Fe_3_O_4_-^(1–3)^ were applied as alum sludge flocculants. All the Fenton’s based composites could extrude sludge water and enhancing its dewaterability. Dehydration and sedimentation performance of alum sludge is enhanced by the improvement in Capillary Suction Time (CST) and Specific Resistance for Filtration (SRF). CSP@Fe_3_O_4_-(2 − 1) based Fenton’s coagulation is revealed the highest CST reduction reached to 75% at the optimal operational conditions of 40 and 400 mg/L of CSP@Fe_3_O_4_-(2 − 1) and H_2_O_2_, respectively at pH 3.0. The results compared with the commercial conditioners such as polyelectrolytes, which results in only 37% CST reduction. In comparison with chemical flocculants, the conditioning process based CSP@Fe_3_O_4_ Fenton’s reaction is ecofriendly since it uses both chitosan and magnetite substances that are environmentally benign materials. Also, the system is oxidizing the high proportion of materials in the sludge. Also, elevating temperature of the sludge showed a negative effect in CST enhancement compared to the room temperature. Further analysis showed that the change of the zeta potential (ζ-potential) of the sludge is changed to the more positive values, and the surface morphology attained bigger flocs than of the raw sludge.

## Introduction

Utmost priority has been received in the whole world to acquire high quality potable water^1^. For this concept integrated technologies have been introduced in producing drinking water in water management industry to achieve such goal. However, at the meantime alum sludge (AS) generation is in rapid increase as a byproduct from potable water production^2^. In Egypt, the Al_2_(SO_4_)_3_ consumed in the water treatment plants is about 365 million tones and the result is massive amounts around million tons of produced aluminum residual sludge is discharged from water works plants^3^. Annual alum sludge generation in China is 2.3-million-ton, United States of America is 2 million ton, Malaysia is 2 million ton and Egypt is 1 million ton; however, it is lesser in the European Countries^4–10^.

Direct discharge of alum sludge into open lands areas is the common criteria for its disposal in some countries. However, such discharge is not the appropriate solution since it contaminates the water streams and soil with massive amounts of such waste. Hence, sludge handling is gaining both academia and industrial sectors’ attention^11^. Consequently, prior to any sludge handling and final discharge, it is essential to minimize its massive volumes, which requires the elaboration of an elevated quality routine for the dewaterability and conditioning enhancement technology.

Currently, an ever-increasing worldwide concern has been achieved for minimizing the alum sludge volume prior to its discharge. Practical alum sludge conditioning introduces various available alum sludge conditioners enhancing its dewaterability performance^12^. Previously, applied breakthroughs involving thermal treatments^13^ or chemical-based conditioners such as cationic polyacrylamide stabilizer^14^ and polyaluminum chloride^15^. Commonly, polyelectrolytes based on organic polymers are the most common technique used as a conditioner for enhancing the dewatering performance^16^. Nevertheless, its popularity, the application of such polyelectrolytes substances has its restrictions and drawbacks, which limits its applications. This is causing a massive cost and environmental burden to the society. Thus, such limitations including the residual polymer remaining in the dewatered and conditioned alum sludge that exhibits a long-standing persistent hazard to the environment and aquatic system^17^. This produced shortcoming arouse urgent requirement for suitable environmentally benign alternative conditioner^18^. Not only, the dewatering performances is associated with dewatering and conditioning chemicals but also associated with the bound water contented in the aluminum-based sludge. Furthermore, with the recent developments an environmentally friendly AOPs, advanced oxidation process, conditioning has been used for waterworks alum sludge residue conditioning as an eco-freindly benign technique^19,20^. More recently, Fenton’s reaction is introduced as superior conditioner for sludge treatments^21,22^.

Progressive oxidation technologies that so called “advanced oxidation Processes” (AOPs) based on Fenton’s oxidation system even it is applied in its solo or as a combined conditioner form has been proven to be a promising candidate as alternative conditioner^23^. In such reaction Fenton peroxidation is based on iron ions that augmented with hydrogen peroxide in acidic medium to produce highly reactive species named “hydroxyl radicals” within the catalyzed decomposition of oxidizing agent such as H_2_O_2_ via iron catalyst. Such highly reactive species “hydroxyl radicals” are the key consistent of decomposing the substances in sludge through strong oxidation and altering the sludge structure^24^. Although, the Fenton’s reagent addition for alum sludge dewatering achieving a high performance, its high cost prohibits its practical use for large-scale levels^25,26^. Form this regard, searching for an economic Fenton’s reagent oxidation technique is attaining the researchers’ attention.

Natural polycationic linear polysaccharide derived from chitin is categorized as a non-toxic polymeric material that is so called “*chitosan polymer*” (CSP). CSP is applied in wastewater treatment facilities, while according to the previous literature cited and reported, it is not used so-far in sludge conditioning specially in alum sludge conditioning performances conjugates magnetite (Fe_3_O_4_) nanoparticles. Such combinations are a catalyst initiator of Fenton’s reaction catalyst. Hence, in this current work-study is the first present the evaluation of CSP@Fe_3_O_4_ as an environmentally benign Fenton conditioner based on natural material and green technology to enhance alum sludge dewaterability. CSP@Fe_3_O_4_ is prepared in various combinations, and the process performance is evaluated by investigating the sludge dewatering ability. System parameters are assessed, and the optimal operating conditions are located. Also, the study revealed a comparison with the commercially available conditioners to evaluate the suggested system performance.

##  Experimental investigation

### Raw alum sludge and chemicals

#### Field sample collection

##### Sludge collection and its characteristics

 Waterworks residuals’ that is signified as aluminum-based waste applied in the introduced investigation is collected from the underflow channel of water works settling and sedimentation lagoon in the Waterworks Plant of largest plant located at Shebin El-Kom City in Menoufia governorate at the north of Egypt. The plant is in the southern part of the city and is categorized as the largest Waterworks plant in such place, and the plant is fed with water from the River Nile reservoir. Aluminum sulphate is used in the plant as coagulant and the treatment capacity is of plant is 40,000 m^3^/day. All collected alum sludge samples are assigned to the laboratory for analysis purposes and kept in acid-washed plastic containers. The characteristics of such raw alum sludge is CST; suspend solid; pH; the turbidity of the supernatant and the sludge cake moisture content are summarized in Table 1.

**Table 1:** Alum sludge and its supernatant properties.

Parameters	Unit	Value
CST	Seconds	31
SRF	m/kg	7.25 × 10^11^
Suspend solid	mg/L	11,850
pH	–	6.6
supernatant Turbidity	NTU	276
Al (mg-Al/g sludge)	mg/L	180
Moisture content of sludge cake	%	97

##### Conditioner

 Natural polycationic linear polysaccharide derived from chitin that named as chitosan is conjugated with magnetite to prepare the nanocomposite material, CSP@Fe_3_O_4_ were synthesized through simple co-precipitation method. Initially, chitin biopolymer is dissolved in drops of acetic acid. Also, various combinations of molar ratios of FeSO_4_⋅7H_2_O and FeCl_3_ are dissolved in distilled water. Afterwards, the solutions are mixed through magnetic stirring while a gradual addition of NaOH solution is supplement to the solution to raise the pH to 10.0 value. Subsequently, the combination is heated for 90 °C within 45 min. The result is a precipitate of CSP@Fe_3_O_4_ composite, which is subjected for oven drying and labelled as CSP@Fe_3_O_4_-(1–1), CSP@Fe_3_O_4_-(2 − 1) and CSP@Fe_3_O_4_-(1–3) regarding the weight proportions of both chitosan and magnetite supplementary in the recipe. The resultant materials are used as conditioner for alum sludge dewatering performances.

##### Further conditioners

 For the object of comparison two types of commercial organic polymers that are signified as anionic and cationic polymer types were applied for alum sludge conditioning tests. The cationic type polymer is commercially named Magnafloc LT-25 (supplied by CIBA Specialty Chemicals Ltd., United Kingdom) possess a molecular weight of 10–15 × 10^6^. Also, Magnafloc FO-4140 is used as the cationic type polymers (supplied by SNF SAC ZACde Milieux, France) have a molecular weight of 5 × 10^6^. The organic polymer conditioners are prepared in a concentration of (0.01%) and added at specific doses.

Anionic charged surfactant material named Sodium lauryl sulfate (SLS), (CH_3_(CH_2_)_11_OSO_3_Na) has a molecular weight of 288.38 g/mol and supplied by Sigma-Aldrich used as conditioner for improving the flocculation and dewatering performance of alum sludge. Sodium lauryl sulfate (SLS) conditioner is added in concentrations of 20, 30, 40 and 50 mg/L.

Inorganic conditioner, Gypsum, CaSO_4_.2H_2_O (calcium sulfate dehydrate) (supplied by Saint-Gobain Gyproc Egypt). CaSO_4_.2H_2_O possess a density of 1,400 kg/ m^3^ is used as a skeleton builder and compared to the various organic conditioners.

### Experimental procedure

**Fig. 1:**
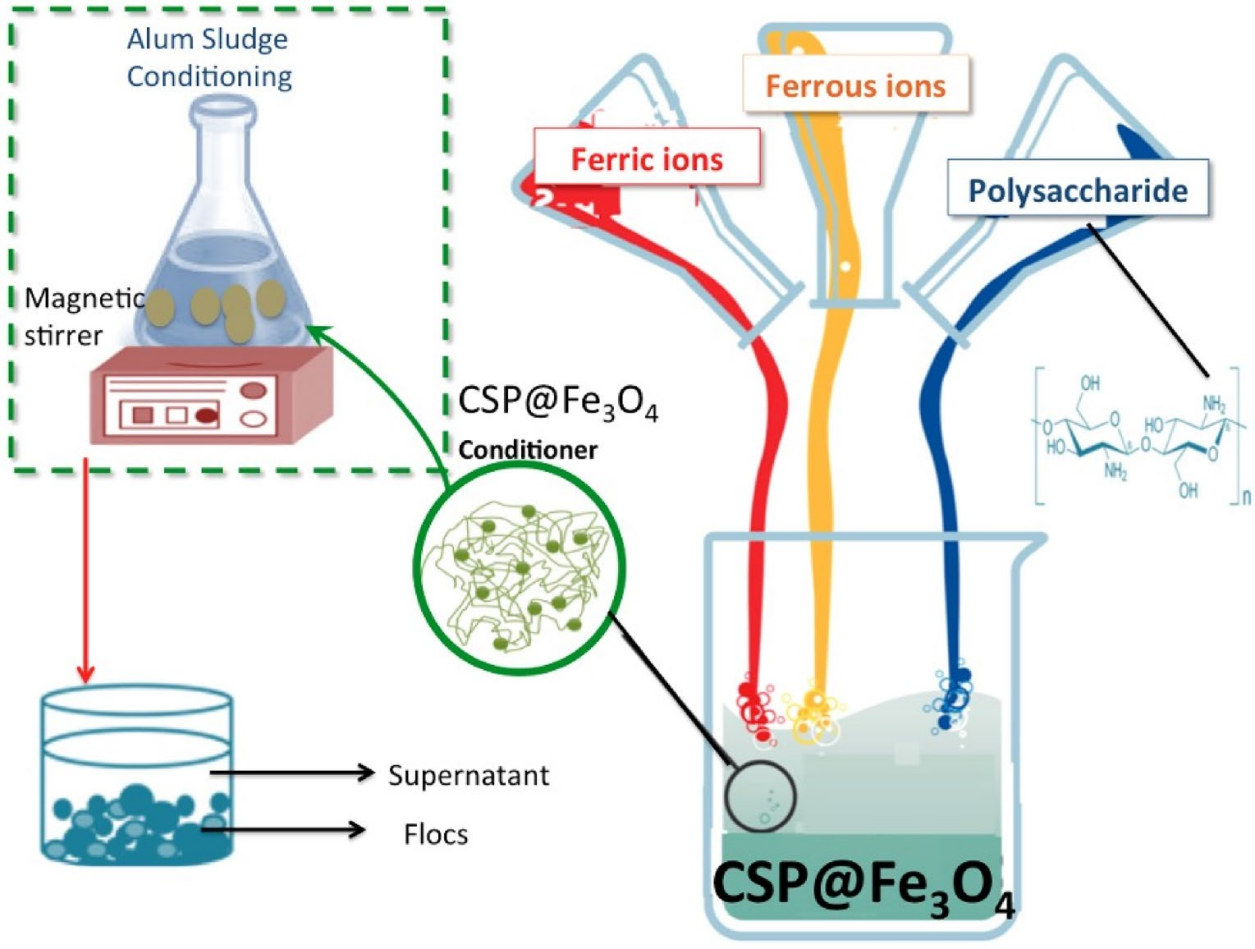
Schematic representation of the preparation of chitosan coated-magnetite conditioner for aluminum residual sludge dewatering.

For the aluminum-based sludge conditioning and dewatering process, AS from the drinking water-works plant is used without further extra chemicals addition. 250 mL of wet sludge is added to 500 mL beaker and jar test is applied. Firstly, pH, if required, is adjusted at the selected temperatures using AD1030, Adwa instrument, Hungary pH-meter. Diluted solutions of H_2_SO_4_ (1:9) and NaOH (1 M) are used for the adjustment of pH. The required doses of CSP@Fe_3_O_4_ composite is then added to the solution as well as 30% w/w H_2_O_2_ peroxide reagent to introduce the Fenton based test and the mixture is subjected for rapid stirring (30 s) followed by slow stirring till the essential time needed. The graphical representation of the CSP@Fe_3_O_4_ composite preparation steps as well as the conditioning experiments are illustrated in Fig. 1.

### Analytical determination

Capillary suction time (CST) before and after conditioning for raw sludge and dewatered sludge, respectively is identified to check CSP@Fe_3_O_4_ performance on sludge dewaterability using Trition CST apparatus (Type 304 M CST). Also, settling test (ST) is conducted according to the standard methods to evaluate the settling speed. Raw sludge and various treated sludge samples were decanted into 100-mL of graduated cylinders to conduct their settling test by detecting their settling behavior. The height of the floc/liquid interface against time of settling was then monitored. Also, the supernatant turbidity of the settled sludge is monitored using ICM turbidimeter. (USA). Also, Chemical Oxygen Demand (COD) for the supernatant were examined according to the standard procedures of sample digestion and Lovibond Checkit direct COD Photometer, Germany for COD analyzer. The total suspended solids (TSS) of sludge supernatant were exhibited subsequent the standard techniques.

The extent of the pH value was explored and adjusted when required by a digital pH-meter (AD1030, Adwa instrument, Hungary).

### Characterization

XRD, X-radiation diffraction graph is applied to investigate the structure of the synthesized material composite. Bruker-Nonius Kappa CCD diffractometer, which is equipped with CuKα radiation at a wavelength of 1.5406 Å was used to investigate such pattern. Also, the morphology of the prepared samples as well as the conditioned sludge were imaged by Field-emission scanning electron microscope (SEM) pictures using FE-SEM, Quanta FEG 250. Such instrument is augmented with energy dispersive spectrum X-ray spectroscopy (EDX) to examine the main oxides contented in raw and conditioned sludge were inspected. Additionally, the particle size distribution was assessed via applying IMAGEJ 1.48 V program. Moreover, the graphs of the Transmission Electron Microscope (TEM) of the prepared samples were investigated by Tecnai G20, FEI machine.

Fourier transform infrared FTIR spectra (Jasco, FT/IR-4100, type A) of the three kinds of the prepared CSP@Fe_3_O_4_ composites were conducted to explore the type of functional group accountable for alum sludge oxidation and dewatering. Also, to assess the impact of CSP@Fe_3_O_4_ composite oxidation on the alum sludge dewaterability performance, the changes in the functional groups of the raw and dewatered aluminum based sludge were recorded.

Zeta potentials (ζ-potential) of raw alum sludge and conditioned/dewatered sludge of these sludge/flocculent aggregates were determined using a Zetasizer (Zetasizer Ver. 6.32, Malvern Instrument Ltd., Worcestershire, United Kingdom) at 25 °C. For each sample, the average of triplicate sample runs was considered. Also, some samples were analysing for floc size distribution.

## Results and discussions

### Characterization of CSP@Fe_3_O_4_ composite

#### X-Ray diffraction structural analysis

**Fig. 2:**
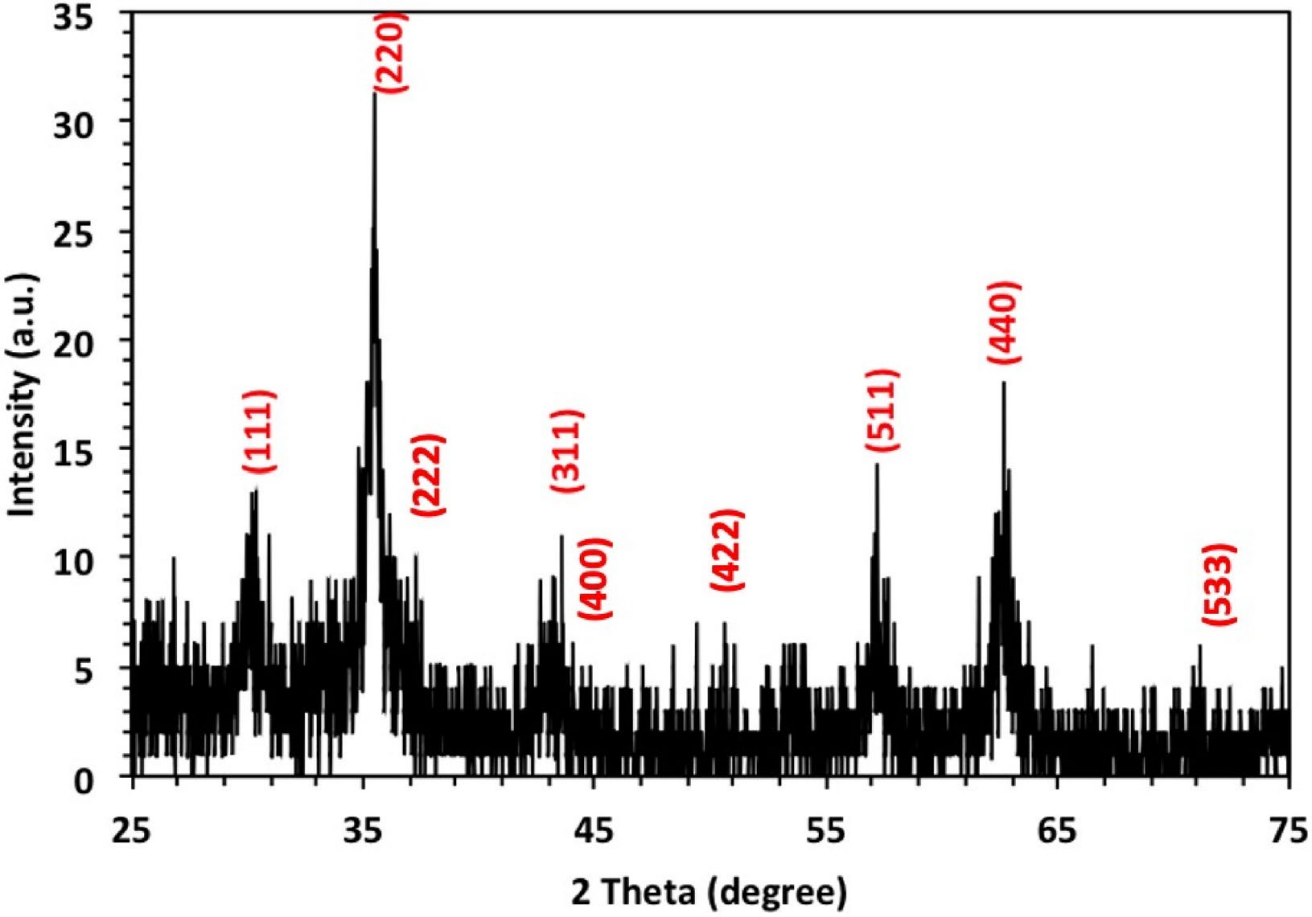
XRD patterns of CSP@Fe_3_O_4_ composite.

X-Ray diffraction patterns of the as-synthesized CSP@Fe_3_O_4_ composite, CSP@Fe_3_O_4_-(2 − 1), is displayed in Fig. 2. XRD data exposes the existence of sharp peaks indicate the presence of the crystalline structure of magnetite as well as the hump of the amorphous biopolymer chitosan. According to the *hkl* planes, the supreme intense peaks of magnetite nanoparticles are allocated as seen in Fig. 2 that assigned with the 2θ of 30.05, 35.49, 37.2, 43.22, 53.63, 57.06, 62.48 and 74.24° that associated with the planes of (111), (200), (222), (311), (400), (422), (511), (440) and (533), respectively. Furthermore, the sharp peak broadening of the magnetite nanoparticles verifies the small particle size of the prepared material. Also, the amorphous nature of biopolymer chitosan is signified in the sample.

#### SEM morphology and particle size distribution of CSP@Fe_3_O_4_

**Fig. 3:**
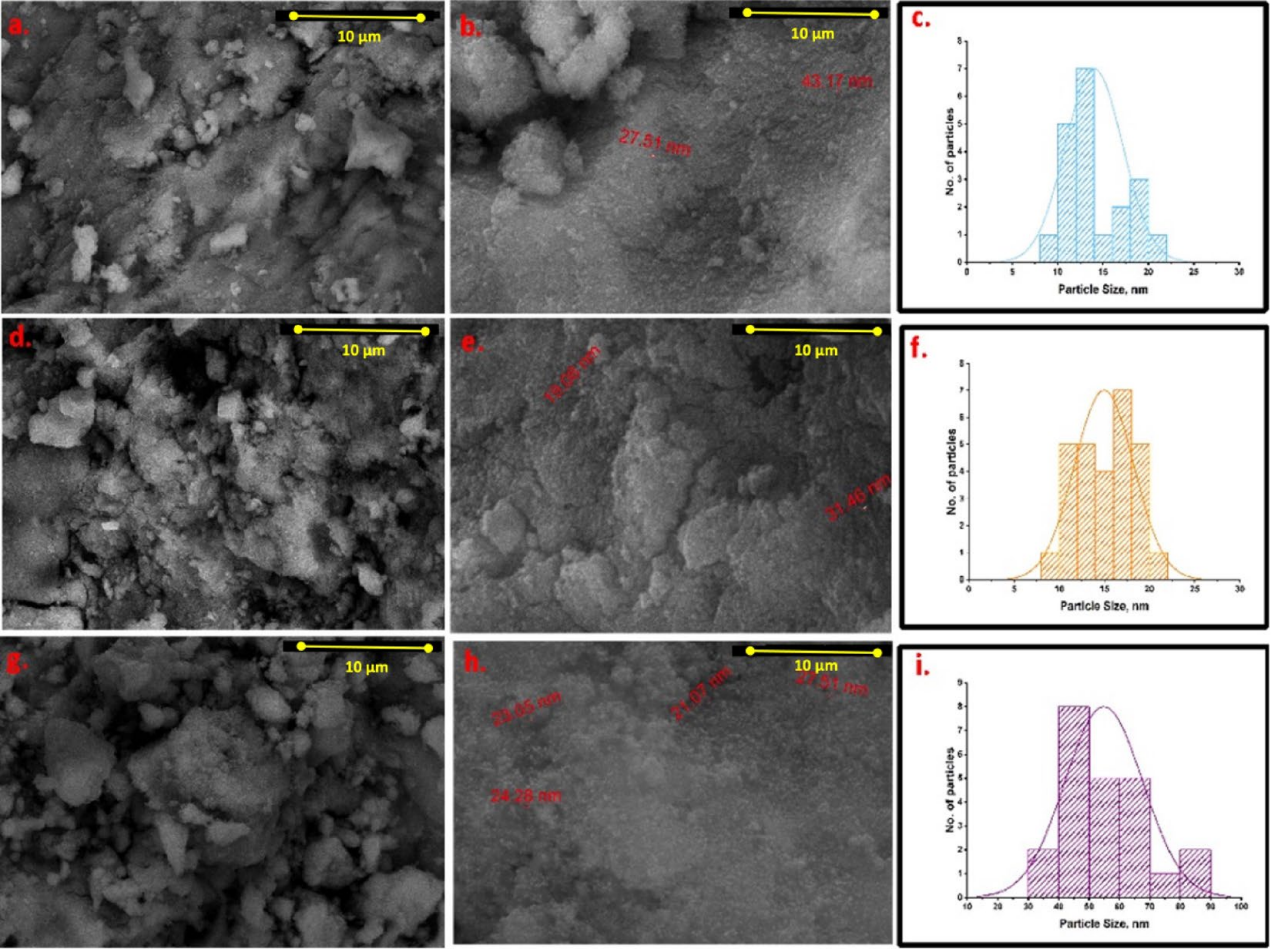
SEM micrograph of varies CSP@Fe_3_O_4_ composites at different magnification (**a**, **b**) CSP@Fe_3_O_4_-^(1– 1)^, (**d**, **e**) CSP@Fe_3_O_4_-(2 − 1) and (**g**, **h**) CSP@Fe_3_O_4_-^(1–3)^ augmented with the particle size distribution histograms of the synthesised (**c**) CSP@Fe_3_O_4_-^1– 1)^, (**f**) CSP@Fe_3_O_4_-(2 − 1) and (**i**) CSP@Fe_3_O_4_-^(1–3)^.

Figure 3 presents SEM micrographs of CSP@Fe₃O₄-(1–1), CSP@Fe₃O₄-(2– 1), and CSP@Fe₃O₄-(1–3) composite nanoparticles, illustrating the incorporation of Fe₃O₄ within the chitosan matrix. The images confirm the successful embedding of magnetite nanoparticles along the chitosan framework, where Fe₃O₄ particles appear as discrete, predominantly spherical domains distributed throughout the polymeric structure. An increase in the visible population density of Fe₃O₄ nanoparticles with increasing Fe₃O₄ loading is evident, indicating a composition dependent dispersion behavior.

Although SEM provides valuable qualitative insights into surface morphology and particle distribution, it should be emphasized that SEM-derived particle size measurements are inherently limited due to particle overlap, agglomeration, and projection effects. Consequently, the SEM images mainly serve to confirm the presence, shape, and relative dispersion of Fe₃O₄ nanoparticles rather than to provide statistically rigorous particle size quantification.

The median particle size values in Fig. 3c, f, and i were obtained by statistical analysis of high-magnification SEM images. Representative nanoparticles were measured using IMAGEJ 1.48 V software to construct particle size distribution histograms, from which the median particle size was determined. This SEM-based analysis is semi-quantitative and is mainly intended to compare relative particle size trends among samples with different Fe₃O₄ loading ratios rather than to provide absolute size values. The particle size distribution histograms shown in Fig. 3c, f, and i reveal median particle sizes of approximately 13.26 nm, 14.78 nm, and 82.80 nm for CSP@Fe₃O₄-(1–1), CSP@Fe₃O₄-(2–1), and CSP@Fe₃O₄-(1–3), respectively. The pronounced increase in apparent particle size at higher Fe₃O₄ loading is attributed to enhanced nanoparticle aggregation within the chitosan matrix, a phenomenon commonly observed in magnetite–polymer composites at elevated inorganic content. Despite the semi-quantitative nature of SEM-based PSD analysis, the observed nanoscale dimensions and compositional trends support the formation of CSP@Fe₃O₄ composites with substantial exposed surface area. This morphological characteristic is favorable for sludge conditioning applications, as it enhances surface interaction, adsorption capacity, and catalytic accessibility during the conditioning process.

For conditioning performance, the nanoscale CSP@Fe₃O₄ composites exhibit a high specific surface area that provides abundant active sites for interaction with sludge components, including extracellular polymeric substances (EPS) and negatively charged colloids. The increased surface area enhances adsorption, charge neutralization, and polymer bridging, promoting floc formation and consolidation. Moreover, exposed Fe₃O₄ sites improve catalytic accessibility during Fenton-like reactions, facilitating EPS oxidation and bound-water release, which collectively enhance sludge dewaterability and conditioning efficiency.

#### TEM images of CSP@Fe_3_***O***_4_

**Fig. 4:**
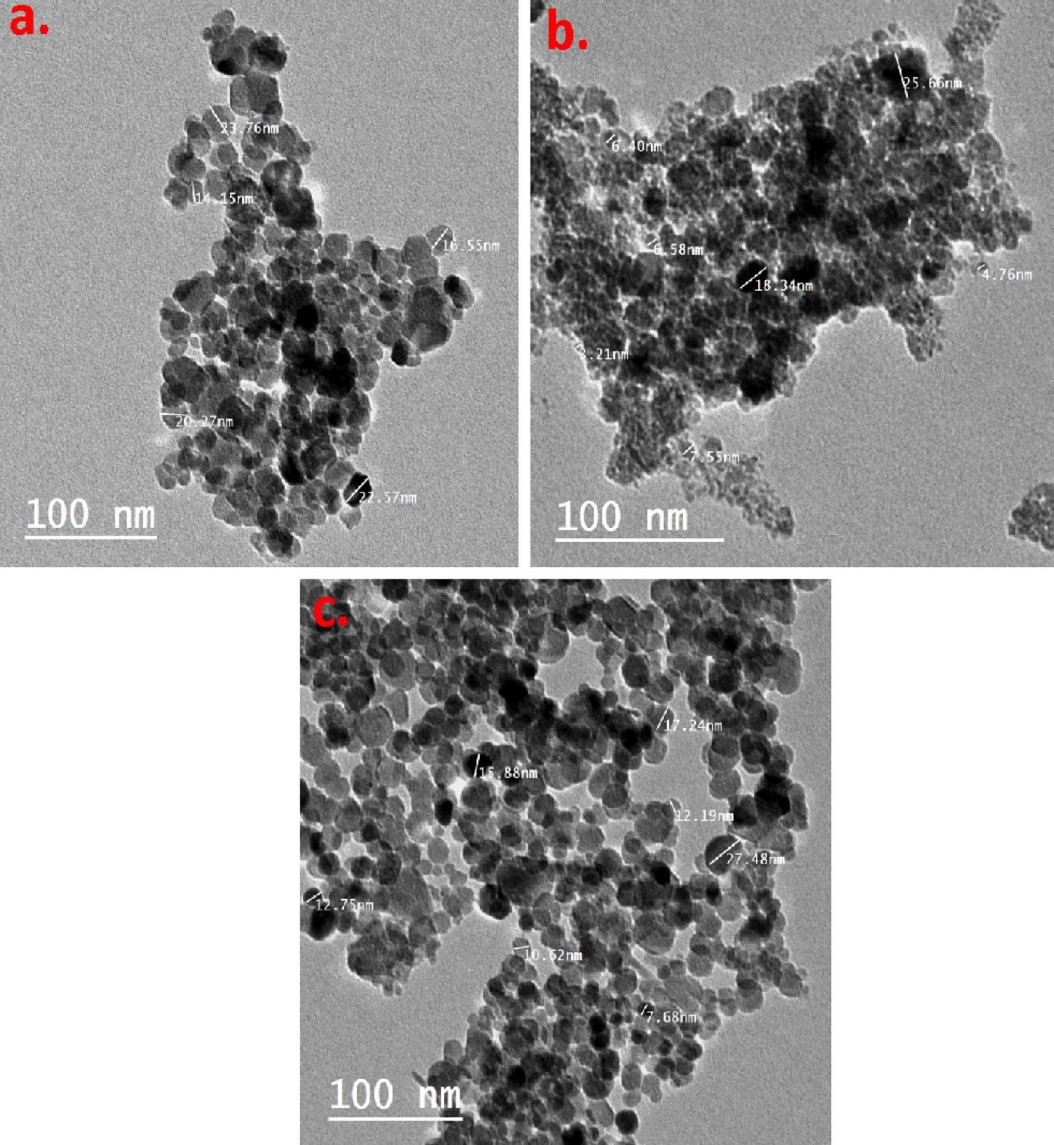
TEM images of various (**a**) CSP@Fe _3_ O _4_-(1–1), (**b**) CSP@Fe_3_O_4_-(2−1) and (**c**) CSP@Fe_**3**_O_4_**-**(1–3) nanocomposites.

To further examine the morphology of CSP@Fe_3_O_4_ nanocomposite, Transmission Electron Microscope (TEM) analysis has been carried out (Tecnai G20, FEI). Figure 4 exhibits the TEM micrograph of polyelectrolyte CSP supplemented magnetite nanoparticles as nanocomposite substances. The TEM micrographs of the composite explore the information morphology of the nanoparticles. It might be investigated from Fig. 4 that the magnetic particles possess a spherical type of shape. The dark dense areas categorize the presence of the crystalline magnetite particles, whereas the bright spots adjacent the magnetite particles are assigned for amorphous CSP substance. But it is signified that in all samples Fig. 4a-c some particles are agglomerated with each other since the magnetite dipole/dipole attraction force. Furthermore, the quantity of dispersed spherical magnetite nanoparticles are differing in each composite CSP@Fe_3_O_4_-(1–1), CSP@Fe_3_O_4_-(2 − 1) and CSP@Fe_3_O_4_-(1–3), in Fig. 4a, b and c due to the various ratios of the magnetite in the chitosan surface.

#### FTIR of CSP@Fe_3_*O*_4_

**Fig. 5:**
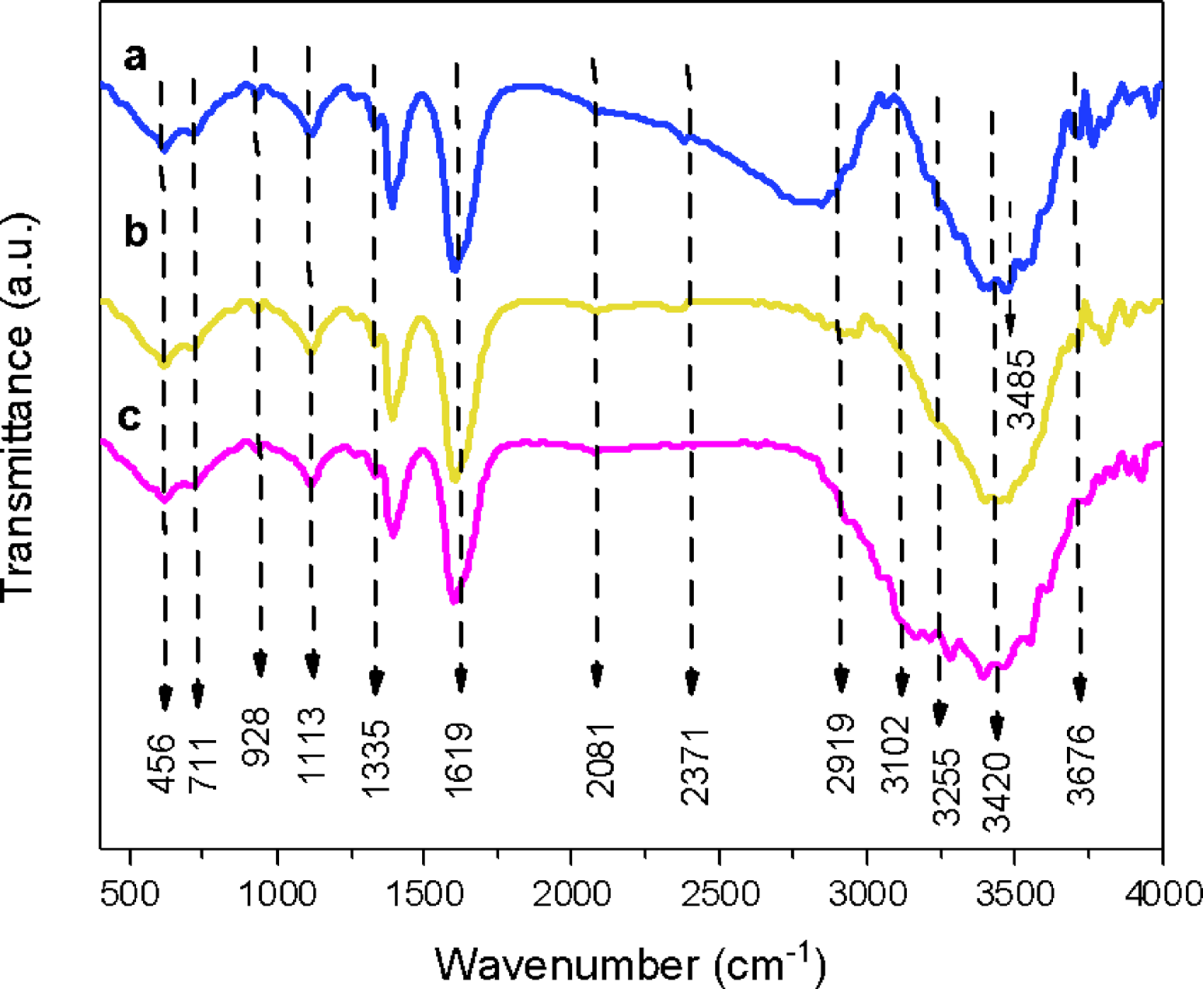
FTIR of spectrum of chitosan-magnetic (CSP@Fe _3_O_4_) prepared by co-precipitation (**a**) CSP@Fe_3_O_4_**-**(1–1), (**b**) CSP@Fe_**3**_O_4_-(2 − 1) and (c) CSP@Fe_3_O_4_**-**(1–3) nanocomposites.

Figure 5 displays FTIR spectra of CSP@Fe_3_O_4_ (a) CSP@Fe_3_O_4_-(1–1), (b) CSP@Fe_3_O_4_-(2 − 1) and (c) CSP@Fe_3_O_4_-(1–3) nanocomposites to validate the iron oxide nanoparticles on chitosan composite and the data represented the spectra between 400 and 4000 cm^− 1^. The graph showed the absorption bands at 3420 cm^− 1^ due to − NH or –OH stretching vibrations and 2371 and 2081 cm^− 1^ for –CH stretching of copolymer of chitosan, respectively^27^. The spectrum of bands at 1335 and 1619 cm^− 1^ are corresponding to − NH bending and C-N stretching which signifies the amide II that verifies the occurance of chitosan biopolymer^28^. However, the existence of iron oxide is represented by the 618 cm^− 1^ peak that corresponded to the Fe − O stretching vibration^29,30^. Additionally, the occurrence of C-O-C verifies the combination between the biopolymer and iron oxide nanoparticles. Additionally, by increasing the magnetite nanoparticles in the composite sample, the CSP@Fe_3_O_4_-(1–3), the result is a weak absorption spectrum of C-O-C. in contrary the sharp absorption band in the case of CSP@Fe_3_O_4_-(2 − 1).

### Alum sludge dewaterability

#### Sludge dewaterability by Fenton’s reaction conditioning

##### Conditioning time of alum sludge with Fenton’s reaction

**Fig. 6:**
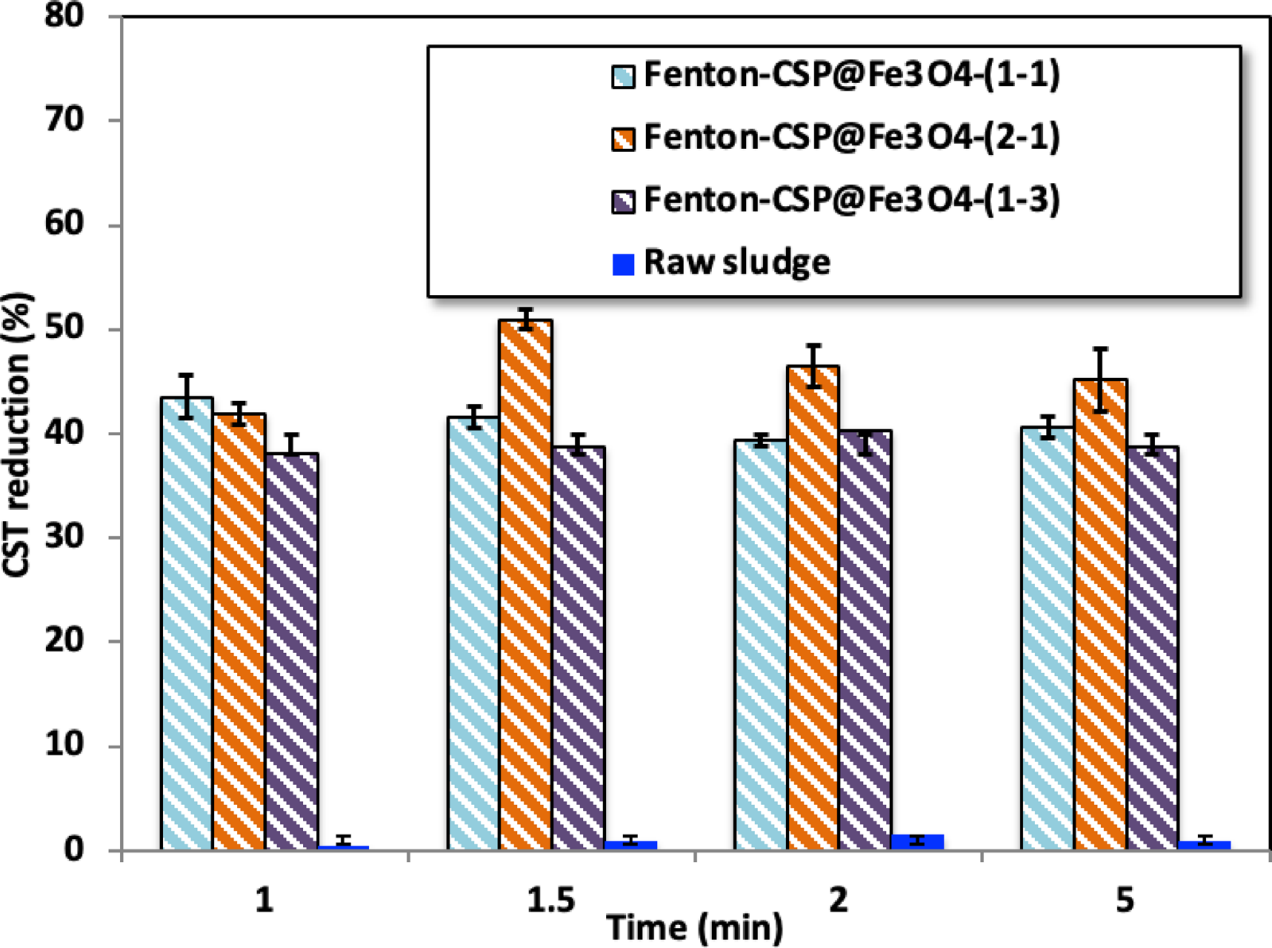
Effects of different CSP@Fe_3_O_4_ composite based Fenton’s oxidation on CST reduction efficiency (%) of alum sludge (Operating parameters: H_2_O2 = 400 mg/L; composite = 40 mg/L; pH = 6.6) and compared with raw sludge.

Figure 6 conjointly explains the influences of various Fenton’s reaction systems based on CSP@Fe_3_O_4_-(1–1), CSP@Fe_3_O_4_-(2 − 1) and CSP@Fe_3_O_4_-^(1–3)^ nanocomposite as a dual polyelectrolyte and Fenton catalyst together to provide dual conditioning of the alum sludge dewaterability and compared with the correspond sludge dewaterability of the raw sludge. Generally, lower CST means better filtration performance^31^. The consequences of Fenton oxidation time on aluminum-based sludge conditioning were assessed at various flocculation time ranged from 1 to 5 min. Various sets of experiments at various CSP@ Fe_3_O_4_ composites as the source of the catalyst and also at varied conditioning time.

The data in Fig. 6 revealed that CSP@Fe_3_O_4_ addition might even result in an elevated CST reduction efficiency representing that Fenton’s oxidation has effective function to enhance sludge dewaterability performances. The optimal CSP@Fe_3_O_4_ Fenton system addition to attain highest CST reduction performance was corresponding to CSP@Fe_3_O_4_-(2 − 1), at which 51% CST reduction effectiveness was attained after 1.5 min of flocculation time. However, the CSP@Fe_3_O_4_-^(1–1)^ and CSP@Fe_3_O_4_-(1–3) revealed 42 and 39% CST reduction, respectively. This reveals the efficacy of Fenton’s oxidation as a substitute conditioner in aluminum water works sludge dewatering. Notably, almost no CST reduction is achieved when the raw sludge is conditioned with time. However, it is noteworthy to mention that lower or higher flocculation time rather than 1.5 min results in a deduction in the CST efficacy for all systems. This in accordance with the previous finding conducted by Mo et al.^32^.

The data, as exhibited in Fig. 6, displays obviously that the maximum CST deduction performances attained in the preliminary dewatering time stage of conditioning time for all Fenton’s cases, whereas with the extended dewatering time, the result is an insignificant upgrading of alum sludge dewaterability. Higher time resembled to a comparative low CST deduction efficacy. Although the reason is still unclear however it might be associated with the size of the floc formation which enhances the dewaterability^33^. As synthetic flocculants based on natural chitosan polymer and magnetite are the sources of the Fenton’s oxidation, the catalyst exhibited significant potency to dewater the alum sludge. Thus, CSP@Fe_3_O_4_ is estimated to be a promising alternative for substitute chemical Fenton oxidation conditioner system sources as a flocculent for alum sludge. Such alternative is signified as an economic and environmentally benign catalyst. Also, CSP@Fe_3_O_4_ showed exclusive superiority in enhancing the degree of aluminum-based sludge conditioning and dewatering performance through Fenton oxidation technique that could not be achieved with traditional Fenton’s sources. However, it is notably to mention that the rapid oxidation time marks it questionable to realize such methodology in real practice application, it exposes the feature of Fenton system.

##### Fenton’s reagent parameters effect on sludge conditioning

**Fig. 7:**
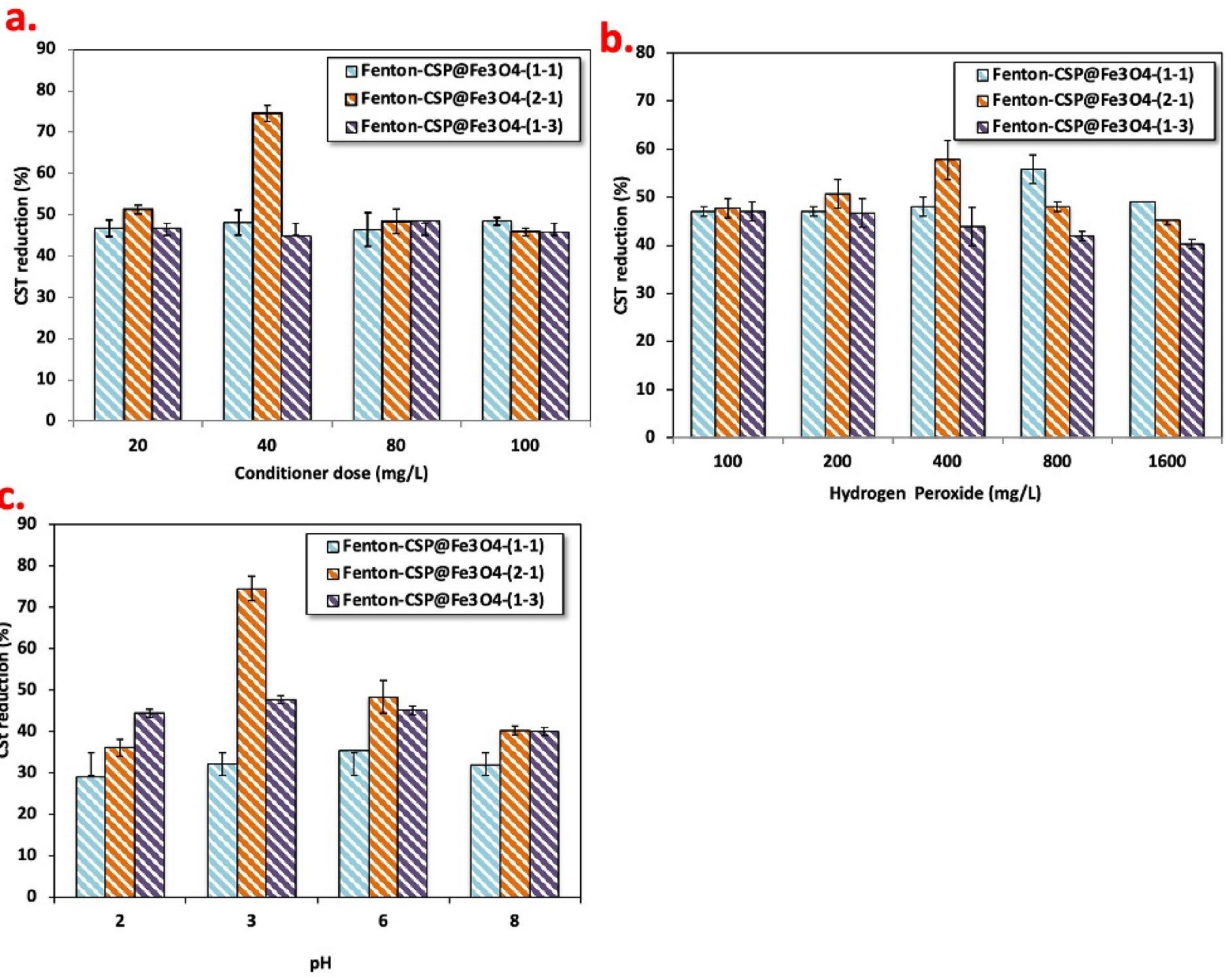
CST reduction efficiency at various Fenton’s reagent operating condtions (Operating parameters: pH 3.0; reaction time is 1.5 min) (**a**) effects of CSP@Fe_3_O_4_ dose; (**b**) effects of hydrogen peroxide concentration and (**c**) effects of various pH values.

Since, Fenton’s parameters influence shows an important role in the alum sludge dewatering and conditioning. Scattered experiments were accomplished to evaluate the influence of different Fenton’s variables effect to locate the optimal working variables that attain best sludge filterability regime. CSP@Fe_3_O_4_ catalyst doses are changed in the three Fenton based systems and the data of such tests are exhibited in Fig. 7a. CSP@Fe_3_O_4_ composite is a rate-limiting stage in Fenton’s reaction cycle. The CSP@Fe_3_O_4_ composite is elevated from the concentration of 20 mg/L into the dose of 100 mg/L and the CST reduction is monitored. The data plotted in Fig. 7a revealed that the significant role of magnetite and chitosan in proceeding the hydroxyl radical’s generation and therefore enhancing the alum sludge filterability^34^. Thus, the CST reduction enhanced from 51 to 75% by increasing the composite dose CSP@Fe_3_O_4_-(2 − 1) from 20 to 40 mg/L. While further elevation in such reagent, results in a deduction in the CST reduction to reach to only 45% rather than enhancing the filterability. It is also notably to mention that CSP@Fe_3_O_4_-(1– 1) and CSP@Fe_3_O_4_-(1–3) composite showing similar trend with a minor effect. This may be attributed by Fe^2+^/Fe^3+^ is hydrolysis in the oxidation reaction and their existence in optimal occurrence attributed in a high and maximum yield of the ·OH radicals’ production that is signified as the dominant dependable of the oxidation test^35^. Also, the ratio of the chitosan/magnetite differs in each sample their presence influencing the ·OH radical generation. To illustrate the non-optimal values of such reagent concentration represented as an ·OH radical inhibitor instead of a generator and declines the coagulating and flocculating reactions^34^.

Figure 7b exhibits the CST reduction rate that is highly affected by the oxidizing reagent “hydrogen peroxide” change. As seen in Fig. 7b, 400 mg/L is leading to 58% CST reduction when the CSP@Fe_3_O_4_-(2 − 1) was used as the initiator catalyst of Fenton’s test. Although, promote increase in the reagent concentration (1600 mg/L), consequences in a decline in the CST deduction effectiveness (45%). This is due to the elevation of aluminum-based sludge conditioners doses, the alum sludge effective tendency (CST) revealed a trend of initial lessening followed by improvement. Although, a minor influence is attained when the CSP@Fe_3_O_4_-(1–1) and CSP@Fe_3_O_4_-(1–3 are applied as the Fenton’s reaction catalyst source. This influence is due to H_2_O_2_ is activating the hydroxyl radicals’ (·OH) generation. Such radicals are the horsepower of oxidation reaction that plays a vital role in the flocculation reaction. H_2_O_2_ amount presence in an optimal concentration has a significant influence on the radicals’ formation and leading to successive reactions. This conducted study is in agreement with the previous finding of Tony and Tayeb^36^ in oxidizing alum sludge using a classical Fenton based solar oxidation test.

It is noteworthy to mention that the increase in hydrogen peroxide (H₂O₂) concentration beyond the optimal level was observed to reduce sludge dewatering performance. This behavior can be explained by the excessive generation of hydroxyl radicals (•OH) during the advanced oxidation process, which can over-oxidize extracellular polymeric substances (EPS) and organic matter within the sludge matrix. While moderate oxidation helps to break down EPS and loosen bound water, excessive oxidation may lead to the formation of smaller, fragmented flocs and increased solubilization of organic matter, resulting in a more viscous and colloidal sludge matrix. Consequently, the sludge retains more water, and the capillary suction time (CST) may increase, indicating deteriorated dewaterability^25^. Therefore, there exists an optimal H_2_O_2_ concentration at which EPS degradation and floc restructuring are balanced to maximize water release, while concentrations beyond this point disrupt floc integrity and impair dewatering performance.

The alum sludge influence on sludge dewaterability performance is illustrated in Fig. 7c. According to the data displayed in Fig. 7c, the Fenton’s reaction based on CSP@Fe_3_O_4_-(2 − 1), the sludge dewaterability improved with the increase in the pH from 2.0 to 3.0 to record 75% CST reduction. But, further elevation in the value of the pH to the alkaline trend, the sludge dewaterability declines since the CST reduction deduced to 40%. However, a minor enhancement in the sludge dewaterability with the use of CSP@Fe_3_O_4_-(1–1) and CSP@Fe_3_O_4_-(1–3), based Fenton’s reaction. Altering the pH values results in a modification of the sludge particles’ surface appearances and characterization besides its definite impact on the extracellular chitosan polyelectrolyte in the aluminum-based sludge. In Fig. 7c, CSP@Fe_3_O_4_-(2 − 1), displayed the best conditioning and dewatering performance.

Through acidic or alkaline circumstances, the colloidal particles of the aluminum-based sludge could be hydrolyzed and thereby, the extracellular polyelectrolytes in the sludge will be dishonored with the relief of the interior water from the alum sludge molecules. Big flocs might be garneted under the achievement of CSP@Fe_3_O_4_-(2 − 1) based Fenton’s system. Nevertheless, the charge conflict between the sludge molecules through acidic circumstances is bigger than that under alkaline environment. As the pH upsurges to the alkaline range, the negative zeta potential elevated continuously. This may be attributed by the high pH value that will elevate the negativity trend of the charge density of the alum sludge and polymeric/iron substance.

However, with the pH control and adjustment, alum sludge could adsorb both ions of H^+^ and/or OH^−^, thus influencing their self-chargeability. Also, the pH might influence on the chitosan extracellular polymeric substances and thereby affect in the sludge filterability^37^. It is noticeably, under the extreme acidic and alkaline medium, the structure of the extracellular chitosan polymer might be decomposed, and the substance elute into the flocculated supernatant. Such phenomenon consequences in an elevation in the organic chitosan polyelectrolyte material in the alum sludge supernatant. Also, chitosan will release into the supernatant under alkaline conditions more than those through acidic medium and the positive charge of chitosan particles will surround the water molecules. Also, the aluminum-based sludge surface charge is a high pH contingent that altered from positive to negative charge. Such investigation presents a considerable data concerning the varying nature of the surface and might influence the electrostatic properties of the alum sludge. Al^3+^ ions is presents in the alum sludge and hexaaquoaluminium ([Al(H_2_O)_6_^3+^) is produced due to the aluminum sulphate coagulant addition^38^. Thus, electrostatic repulsive force in the alkali conditions increases due to the positive charges of the molecules^39^. Thus, the flocs formation is favorable at pH value of 3.0.

Zeta potential measurements were conducted to investigate the surface charge behavior of sludge particles under varying pH conditions. As the pH increased to the alkaline range, the zeta potential of the sludge became more negative, reaching approximately − 28 mV at pH 9. This increase in negative surface charge enhances electrostatic repulsion between individual particles, which could potentially inhibit flocculation. However, in the presence of conditioners such as CSP@Fe₃O₄ or polymeric flocculants, particle bridging and charge neutralization mechanisms dominate, promoting the formation of larger, more compact flocs. These findings demonstrate that the rise in pH to alkaline conditions, as reflected by zeta potential data, is consistent with the observed flocculation behavior and supports the claims regarding enhanced sludge aggregation and improved dewatering performance.

It is noteworthily to mention that the acidic pH (3.0) was mainly applied to investigate the maximum catalytic efficiency and mechanistic behavior of the CSP@Fe₃O₄ nanocomposite under the Fenton’s reaction conditions. In practice, pH adjustment can be achieved using low-cost acids or recycled acidic streams, while the conditioned sludge retains catalytically active iron species and can be reutilized as a secondary material for wastewater treatment under acidic Fenton oxidation systems, partially offsetting pH adjustment costs.

##### Temperature effect on Fenton’s conditioning

**Fig. 8:**
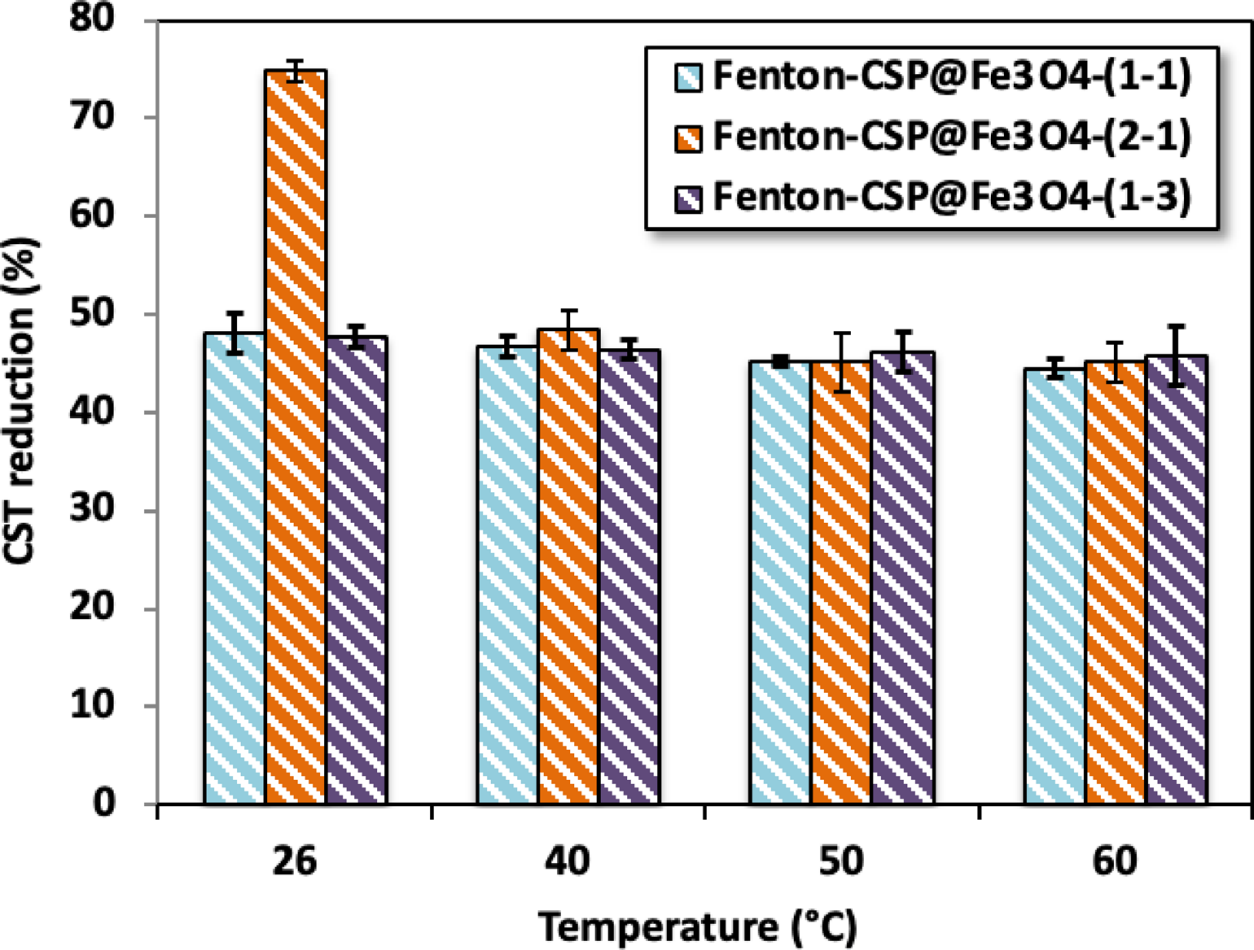
Temperature effect on alum sludge dewaterability by Fenton reaction.

Commonly, Fenton reaction of oxidation is conducted at room temperature. Although, increasing the temperature could also influence the conditioning system. The data of dewaterability that exhibited the enhancement by alum sludge conditioning using Fenton reagent at varied sludge temperatures above the range of 26° to 60 °C are demonstrated in Fig. 8. The conditioning is conducted using CSP@Fe_3_O_4_ 40 mg/L and 400 mg/L, at pH 3.0.

The trends in Fig. 8 designate the insignificant influence of elevating temperature in the Fenton reaction based CSP@Fe_3_O_4_-(1– 1) and CSP@Fe_3_O_4_-(1–3) catalyst during aluminum based sludge conditioning since CST reduction as a filterability indicator is declined from 48% to 44% and from 47% to 43% when CSP@Fe_3_O_4_-(1– 1) and CSP@Fe_3_O_4_-(1–2) is used as a catalyst, respectively, with the temperature elevation from room temperature to 60 °C temperature. Compared with the values of 75 to 45% with the elevation from room temperature to 60 °C temperature, respectively when CSP@Fe_3_O_4_-(2 − 1) is applied. Hence, the concept of its influence on the aluminum-based sludge conditioning/dewatering is consequential from the Fenton’s oxidation system, instead of the thermal effect. In addition, the thermal effect has a negative influence impact on alum sludge conditioning performance. Such investigation might be predictable from the phenomenon of hydrogen peroxide is consumed with the temperature increase^40^. Therefore, for the regard of attaining a satisfied filterability of the alum sludge, the temperature is kept at ambient temperature. This investigation is not in agreement with the previous findings of^41^ who stated a significant improvement on activated sludge dewatering characteristics when the temperature was elevated.

#### Sludge dewaterability by inorganic conditioners

**Fig. 9:**
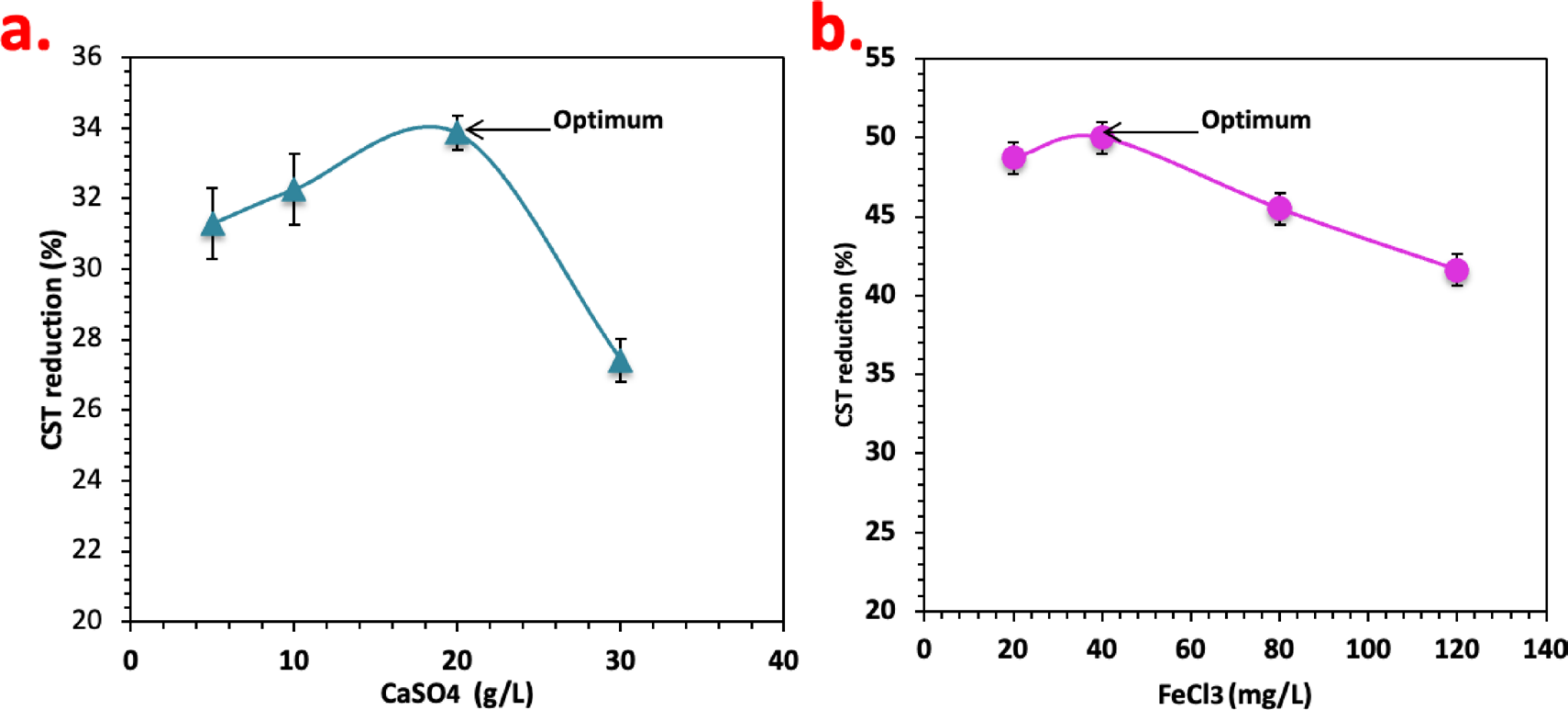
Effects of (**a**) CaSO_4_ and (**b**) FeCl_3_ on alum sludge dewatering performance.

Inorganic compounds including calcium sulfate dehydrate that is signified commercially as Gypsum (CaSO_4_) as well as Ferric chloride (FeCl_3_) conditioning on the alum sludge dewaterability were investigated by measuring the CST of the conditioned/dewatered alum sludge as displayed in Fig. 9. The CST of the raw alum sludge exhibiting poor dewaterability, however, an increase in the inorganic conditioner dosage from 0 to 20 g/L for CaSO_4_ and from 0 to 40 mg/L for FeCl_3_ significantly improved the sludge dewaterability to reach to 34 and 50 CST reduction after using CaSO_4_ and FeCl_3_, respectively.

CaSO_4_ was applied as skeleton builder for the object of affording comparative information with the Fenton based chitosan polymer conditioner. Notably, it is important to conclude that the presence of CaSO_4_ assists to construct a superior porous, penetrable and inflexible lattice structure of the dewatered alum sludge cake^42^. Nevertheless, in the case of the FeCl_3_ conditioner creates crystalloids clumps on the flocs’ external surfaces, which helps in water withdrawal from the sludge^43^.

The alum sludge dewaterability performance changed and altered to be poor as the inorganic conditioners concentration were continued to add into the sludge sample. The sludge dewatering performances were reduced to only 27% where the CaSO_4_ dosage escalated to 30 g/L. Also, the CST reduction recorded only 41% when the FeCl_3_ amount elevated to 120 mg/L. Thus, the optimum conditioners dosages are 20 g/L and 40 mg/L CaSO_4_ and FeCl_3_. This research was similar to the current investigation of Fenton treatment in the sludge dewatering, the lower doses of the conditioner results in an improvement in the alum sludge dewatering performances but the higher doses were the opposite^44^.

#### Sludge dewaterability by organic conditioners

**Fig. 10:**
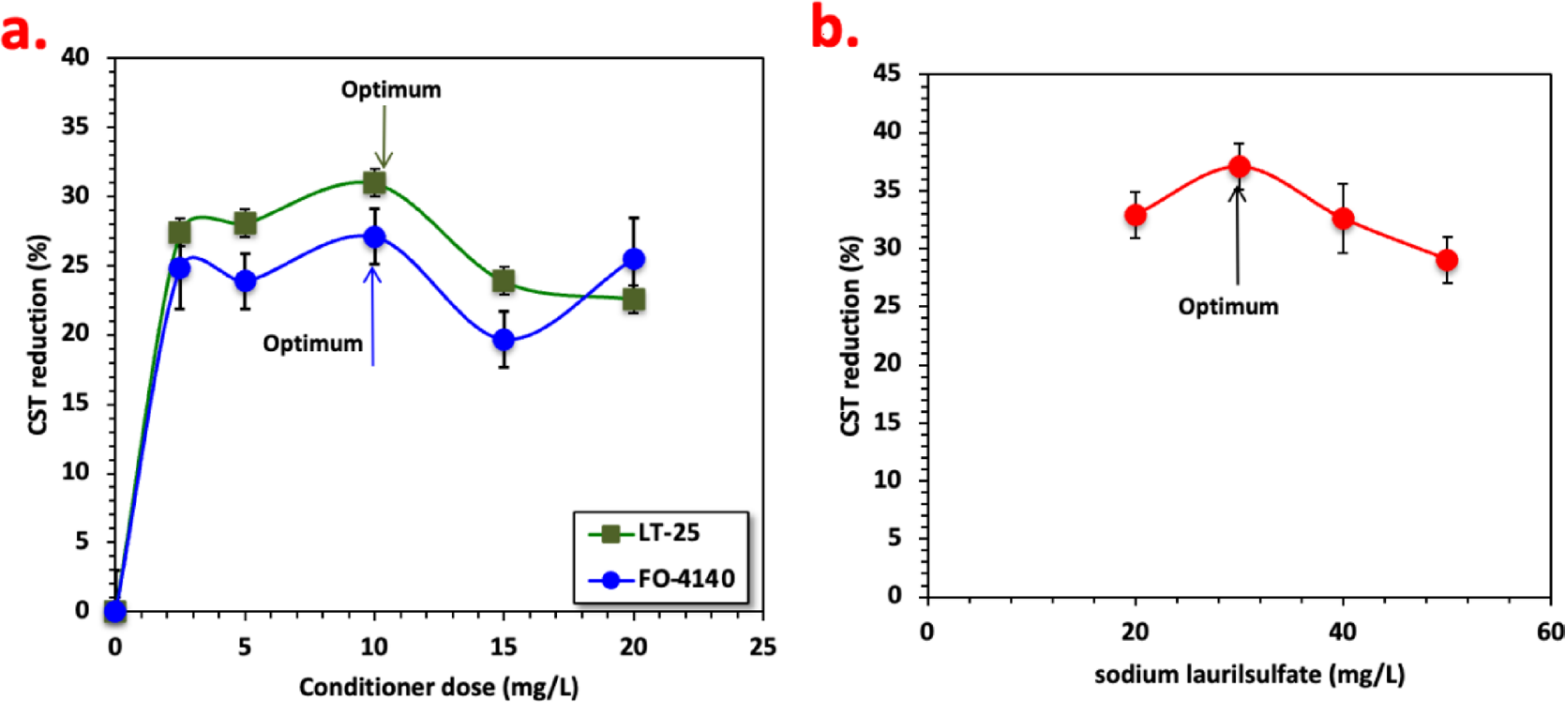
Effect of various inorganic conditioner (**a**) cationic and anionic polyelectrolytes and (**b**) anionic surfactant on alum sludge dewatering performances.

For the object of comparing the traditional conditioners with the current work, the effectiveness of three organic conditioners namely cationic and anionic polyelectrolytes as well as anionic surfactant is assessed to evaluate their effect on alum sludge dewatering performances. Figure 10a and b depicts typical results for the conditioner dosage advancement on the sludge dehydration performance and floc formation via CST reduction. From the data displayed in Fig. 10a, it is clear that both polyelectrolytes have positive effects on the overall sludge filterability performance, but the dosages of both polyelectrolytes get different CST correlations. Also, the anionic magnafloc polyelectrolyte conditioner (LT-25) depicts more pronounced effect than cationic polyelectrolyte conditioner (FO-4140) on the CST reduction accomplishment. The water contented of the conditioned sludge is slightly declined by the elevation of both polyelectrolytes amount from 2.5 to 10 mg/L. However, extra upsurge in the polyelectrolytes’ conditioners dose results in a decline in the CST reduction effectiveness. The danger in adding excessive polyelectrolyete conditioner amount is not merely the extra conditioning cost in reagents, but also the dewatering performance will become ineffective.

Polyelectrolyete conditioning results in a decline in the tendency of water content of the dewatered sludge. This is attained slightly after polyelectrolytes addition due to the floc formation and the difference in the alum sludge cake porosity. Also, the sludge cell walls might be destroyed after the polyelectrolytes addition, which is then followed by the water release inside the cells. It is noteworthy to mention that the FO-4140 cationic polymer contained positively charged hydrophilic groups like play a serious role in the hydrophobicity of the dewatered aluminum-based sludge surface. However, oppositely LT-25 has a negative charge hydrophilic groups that utilized an adverse influence on the dewatering effectiveness of the alum sludge. Therefore, the dewaterability of the alum sludge after LT-25 conditioning was accordingly improved.

By comparison, the CST reduction efficiency anionic sodium lauryl sulfate (SLS) surfactant conditioner is evaluated and Fig. 10b depicts typical results for the conditioner dosage evolution on the CST deduction performance. The experimental data in Fig. 10b exposes the consequence supplement of the surfactant on enhancing the alum sludge conditioning/dewatering attitude. Mixing the surfactant with the alum sludge results in a deduction on the volume of dewatered sludge since increasing the SLS dose from 20 to 30 mg/L. This enhances the dry solid content attains the floc formation. The CST reduction attained reached to 37%. However, no superficial sludge-liquid departure interface is poorly recognized with the high concentration of the SLS (50 mg/L) as the surfactant simply endorsed dewatering and failed to bridge the alum sludge floc molecules^45^. Thus, the produced sludge flocs in the medium are very minor in size, loose and fragile.

SLS is amphiphilic organic compounds, which means it possess both hydrophobic groups and hydrophilic groups. The accumulation of extra SLS produces further negative ion charge and produced the considerable discharge. Generally, chemical-based conditioners increase sludge dewaterability by various mechanisms. For instance, SLS molecules may be absorbed onto the alum sludge solid’s surface through various ion exchange and ion pairing as well as hydrogen-bonding besides the presence of Van der Waals force and hydrophobic attachment prior to it exceeds the critical limits^46^.

#### Comparison of different organic “Fenton’s and polymer” conditioners on chemical characteristics and sludge morphologies

##### Conditioning/ treatment efficiency and characteristics

**Fig. 11:**
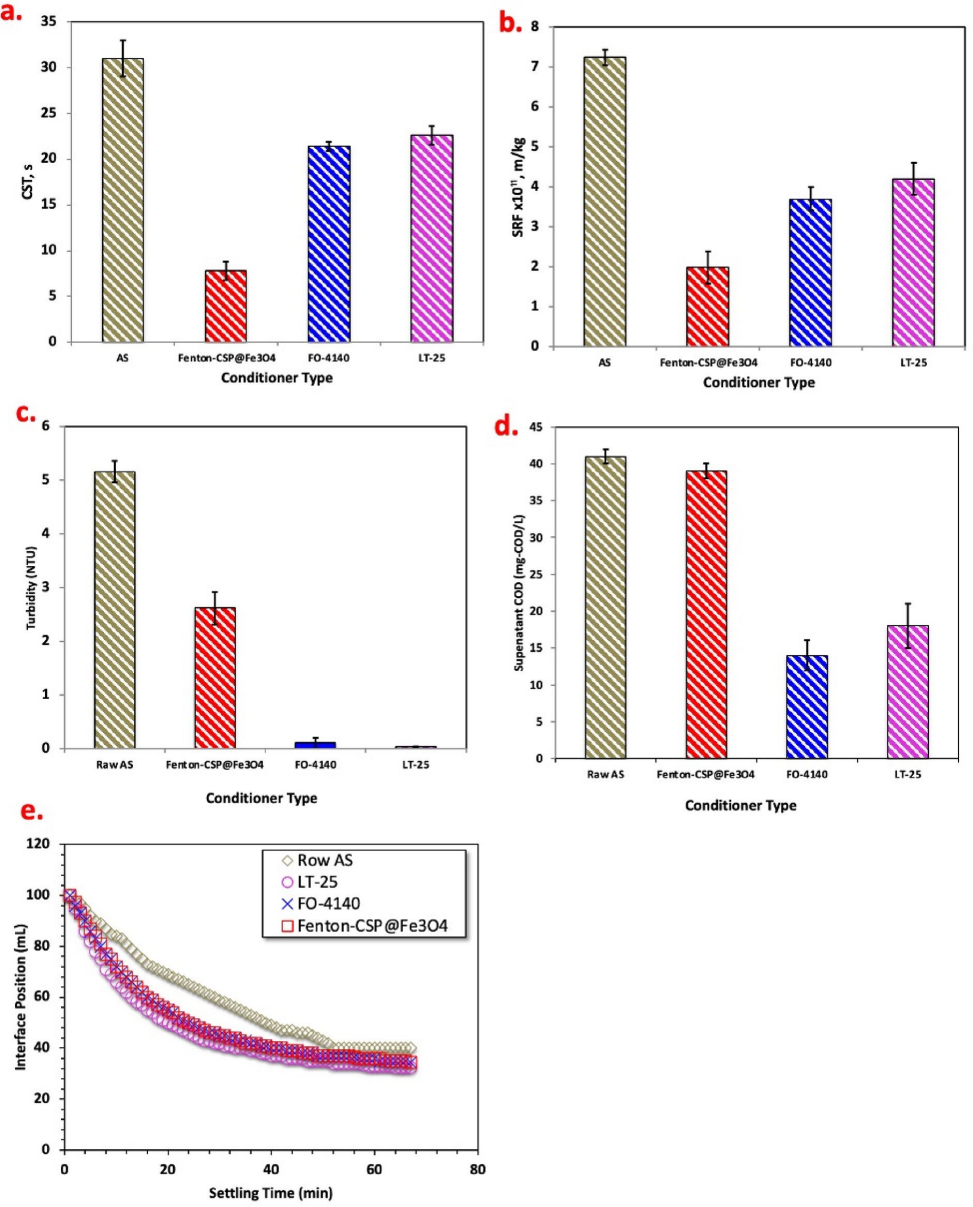
Raw and conditioned alum sludge characteristics (**a**) CST (**b**) Settling time, (**c**) Turbidity of the supernatant and (**d**) Supernatant COD.

For the object of comparing the dewatering data, the CST filterability indicator value was assessed at the optimum added dose of the various sludge conditioners tendency were compared besides their settling tendency, the supernatant turbidity and chemical oxygen demand (COD). The comparative results of conditioning with Fenton’s conditioner and organic polymer conditioning are explored in Fig. 11 that exposed the consequences of sludge dewatering under various conditioners. CST was used to evaluate the sludge dewatering performance. The data displayed in Fig. 11 (a) exhibited that the dewatering capacity of the raw sludge was poor, and its CST was 31 s. When dosages of Fenton (CSP@Fe_3_O_4_-(2 − 1) 40 mg/L and H_2_O_2_ 400 mg/L), CST was the smallest 7.8 s of the best dewatering performance that signifying 75% reduction. Meanwhile, with the supplement of cationic Magnafloc FO-4140 and anionic Magnafloc LT-25 polymers, the sludge is obtainable the dewatering performance of 21.4 and 22.6s, respectively which is better than that of the raw sludge. The alum sludge dewatering data presented adding of conditioners was constructive to sludge dewatering, but not all attain the best possible dewaterability.

The reduction in capillary suction time (CST) is further supported by the relative sludge filterability (RSF) results, as shown in Fig. 11b. A lower CST corresponds to a reduced resistance to water flow through the sludge cake, which is directly reflected by improved RSF values. In particular, the specific resistance to filtration (SRF) decreased from 7.25 × 10¹¹ m/kg for raw alum sludge to 1.99 × 10¹¹ m/kg, representing a 72% reduction when the Fenton-based CSP@Fe_3_O_4_-(2−1) conditioner was applied. In comparison, SRF values were reduced to 3.7 × 10¹¹ m/kg and 4.2 × 10¹¹ m/kg upon conditioning with cationic Magnafloc FO-4140 and anionic Magnafloc LT-25, corresponding to 48% and 42% reductions, respectively. Thus, the observed decrease in RSF after conditioning indicates enhanced permeability of the sludge matrix, which can be attributed to the formation of larger and more compact flocs. These structural changes create more effective drainage pathways, facilitating faster water release during filtration. Therefore, the RSF results corroborate the CST measurements and confirm that sludge conditioning improves dewaterability primarily by reducing filtration resistance rather than by significantly lowering the total moisture content^25,33^.

In the case of using Fenton’s reagent based CSP@Fe_3_O_4_-(2 − 1) conditioner, the enhancement in the sludge filterability and dewaterability is correlated with the highly reactive radicals (OH). Such radicals might attack the cells of the organic molecules of alum sludge. Thus, such reaction is leading to the elevation of the sludge hydrophobicity and therefore the interstitial water trapped on the organic particles of the sludge is released^38^. To add up, magnetite including dual iron ions which also affects and enhances the sludge flocculation and coagulation. Chitosan also presented in the CSP@Fe_3_O_4_-(2 − 1) sample as the source polyelectrolyte, which previously recorded a good flocculation enhancement capability. Such hybrid combinations play a energetic function in the effective alum sludge conditioning and dewaterability. It is noteworthy to mention that chitosan, as a conditioner of polyelectrolyte is an environmentally benign polyelectrolyte source. In the case of commercial polymers dewatering, the conditioning reaction mechanism differs since cationic Magnafloc FO-4140 and anionic Magnafloc LT-25 polyelectrolytes are serving as a role of charge neutralization and inter particle or might be primary flocs bridging. However, the different ionic charges of the two polymers, cationic and anionic imitate their adsorption tendency onto the alum sludge molecules^47^.

Figure 11c provides the supernatant turbidity prior and after conditioning processes. The turbidity was the lowest at around 0.04 NTU when polyelectrolyte conditioning type is used especially anionic Magnafloc LT-25 followed by 0.11 NTU for cationic Magnafloc FO-4140. This might be due to the presence of exact amount of polyelectrolyte to form flocs and coagulate the organics from escaping into the bulk solution. However, using Fenton based CSP@Fe_3_O_4_ attaining 2.62 NTU of supernatant turbidity. This might be attributed by the deterioration in the clarity of the supernatant. However, it is notably to remark that the supernatant turbidity after Fenton’s oxidation is still lower than that of the raw sludge after settling behavior (5.16 NTU). This confirms the role of various conditioning in comparison to the raw sludge. However, the reason regarding the high supernatant turbidity for the Fenton coagulation compared to polyelectrolyte conditioners is still unclear. But it may be suggested that the release of iron from the composite onto the water may be a reason. Also, CSP@Fe_3_O_4_ might act as a leaching agent since it is a good adsorbent material. Thereby, the elements augmented with the CSP@Fe_3_O_4_ composite are released into the sludge water. However, this current investigation seems to oppose the distinguished model of Sezgin et al.^48^ that state the supernatant overhead a settling sludge interface is reasonably clear in a bulking sludge.

It recommends that the supernatant COD features for the dewatered/conditioned sludge with Fenton’s test are alike to the corresponding of the raw sludge as displayed in Fig. 11d. This may be explained by the insignificant material release into the sludge supernatant. However, it is better than that of the raw sludge since Fenton system instantaneously react with the organics in the alum sludge thereby oxidizing them. Also, the polyelectrolyte coagulates the sludge and the supernatant become clear. COD results agreed with the supernatant turbidity comparison.

The settling behavior of the sludge samples was evaluated using the settling test, with results presented in Fig. 11e, which tracks the position of the sludge/supernatant interface over a 70-minute period. Conditioned sludge samples exhibited a markedly faster settling rate compared to the raw sludge. Specifically, the interface of conditioned samples is reached within 70 min. This observation demonstrates that conditioning substantially enhances sludge dewaterability. The improvement in settling can be attributed to the formation of larger and more compact aluminum-based flocs during conditioning. The flocculation process increases both the size and density of the aggregates, promoting faster sedimentation. In contrast, the raw sludge is composed of smaller, loosely bound particles that settle more slowly due to lower effective density and weaker interparticle interactions. These findings suggest that the application of conditioners modifies the microstructure of sludge, facilitating the aggregation of particles into floc-like structures, which accelerates settling and improves the efficiency of subsequent dewatering processes. Overall, the settling test confirms a clear correlation between conditioning-induced floc growth and enhanced sedimentation performance, supporting the role of floc morphology as a key factor in sludge treatment.

##### Sludge surface micromorphology

**Fig. 12:**
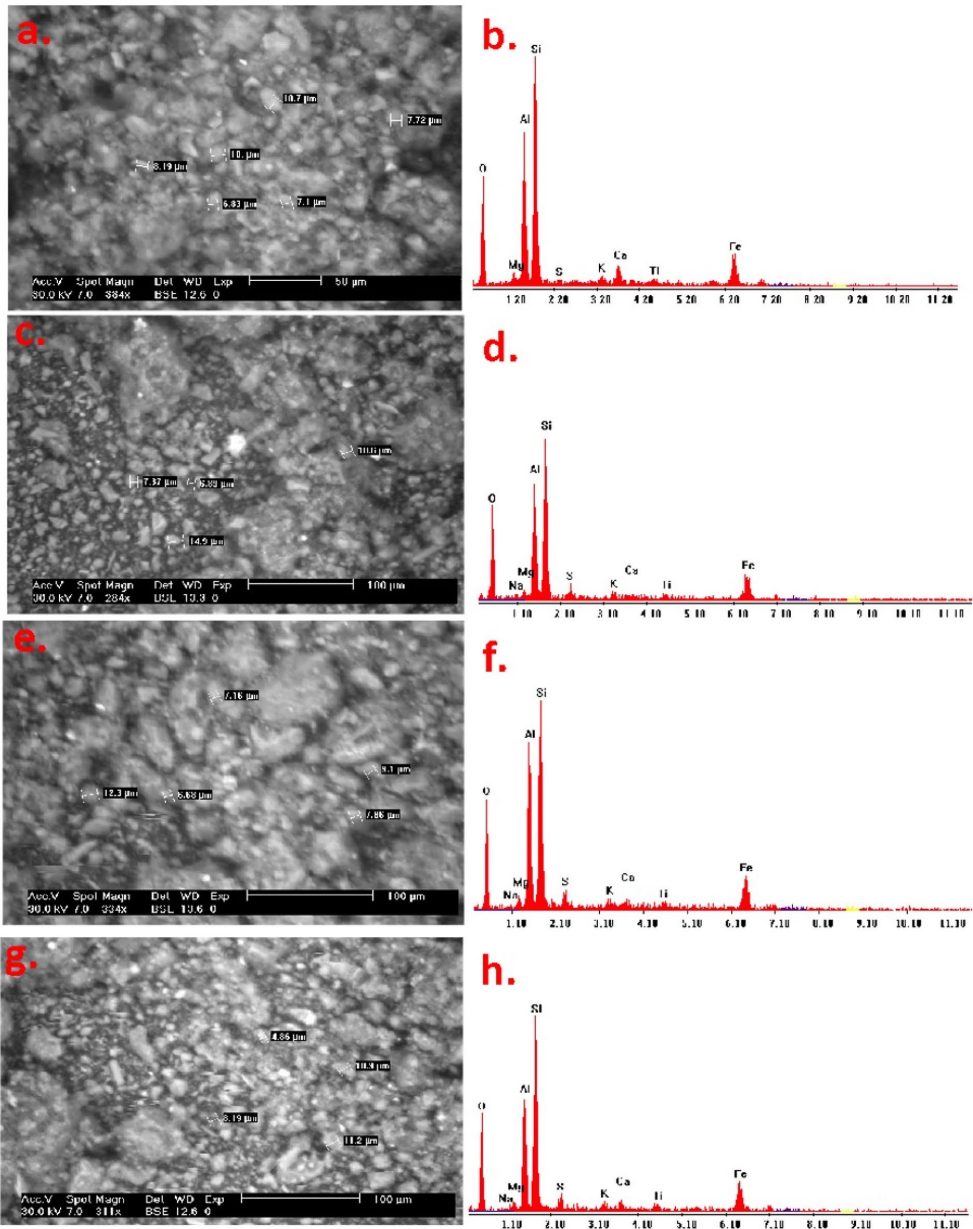
SEM images of raw (**a**) and conditioned alum sludge with various conditioners (**c**) with CSP@Fe_3_O_4_, (**e**) FO-4140 and (**g**) LT-25 augmented with EDX spectra of the each sample (**b**, **d**, **f** and *h*), respectively.

SEM-EDX data according to SEM analysis augmented with EDX as pertained to probe the surface micromorphology of the raw and conditioned sludge via various conditioners. The raw and conditioned sludge cake differs since the former flocs were relatively small and latter was compact. The raw sludge possesses a floc structure that seems to be loosely packed. Figure 12 (a) displayed the raw alum sludge prior conditioning was arranged in a lamellar structure. The sludge surface was a comparatively smooth surface and non-porous structure. In contrast, as exhibited in Fig. 12 (b), there was a change of particle size in the raw and conditioned sludge, where the particle size of the conditioned sludge is increased. This is due to floc surface by redox reaction through Fenton conditioning (as depicted in Fig. 12 (c)) or through polyelectrolyte conditioning as seen in Fig. 12 (e and g), which cause floc rupture. Polyelectrolyte augmented magnetite as a source of Fenton’s reaction in the presence of hydrogen peroxide led to particles agglomeration and the particle size increased. Figure 12 (c, e and g) can be observed that the conditioned and dewatered sludge exhibited pronounced structures and exposed more surface pores that facilitate the water release through more channels, and this improved the sludge dewatering performances. Additionally, the resulting conditioned sludge structures agreed with the fact that the conditioned sludge cake with superior flocculants was mainly attained by bridging with adjacent colloids. Additionally, it seemed that the outflow of intracellular conditioner substances clogged the pores on the flocs of the sludge surface that is signified as another reason for altering the sludge surface.

The electron dispersive spectroscopy (EDX) analysis of raw alum sludge and conditioned alum sludge are displayed in Fig. 12d, d, f, h. As shown in the graphs, these particles indicate the presence of Fe, Al, Si and O composition in the all conditioned and raw sludge. Si was the most predominated element that recognized in all the raw and conditioned sludge samples.

##### FTIR of the sludge cake

**Fig. 13:**
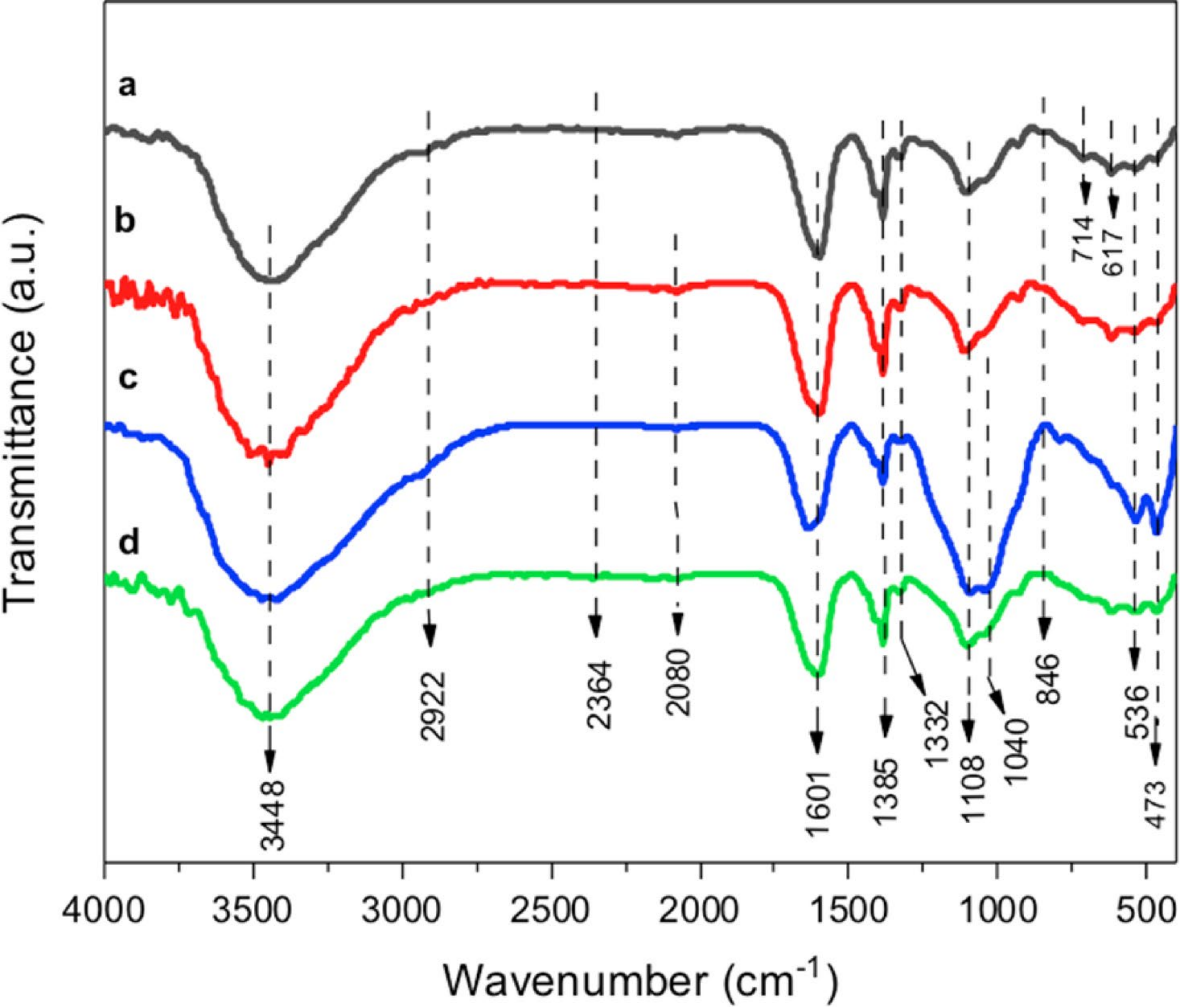
FTIR spectra of (**a**) images of raw and conditioned alum sludge under different conditioners: (**a**) raw alum sludge, (**b**) Fenton conditioned sludge using CSP@Fe _3_ O _4_ -(1–1); (**c**) Fenton conditioned sludge using CSP@Fe_3_O_4_-(2 − 1) and (**d**) Fenton conditioned sludge using CSP@Fe_3_O_4_**-**(1–3).

To further well understanding the effect of the conditioners on alum sludge, FTIR of pure sludge version and various conditioned/dewatered alum sludge cake were investigated to check the change on the functional groups. For all the studied trails (Fig. 13 (a, b, c and d), the broad absorption band of 536 cm^− 1^ is attributed to the stretching vibrations of Al–H-OH, confirming the formation of aluminum hydroxide. The absorption bands at 473 and 847 cm^− 1^ are for O-HSi-HO and Si-OH, respectively^49,50^. Also, absorption bands at 1108 and 1095 cm^− 1^ are for Si-O-Si and Si-OH-H, respectively^19^. As exhibited in Fig. 12a, the absorption peaks at 2857 and 2963 cm^− 1^ are attributed to the CH stretching vibration peaks. The absorption peak at 3448 cm^− 1^ is assigned to the stretching vibration absorption peak of OH in the methyl group^51,52^. The strong stretching vibration absorption signal of C = O is in the region is related to the absorption band of 1601 cm^− 1^. Also, it is observed a weak absorption band at 2080 cm^− 1^ wavelength is reported in Fig. 13b, c and d were caused by the stretching vibrations of N-H bond.

The symmetric sharp signal that is located at a wavelength of 1385 cm^− 1^ wavelength is representative the existence of CH_2_. Accordingly, in Fig. 13a, b, c and d, the weak absorption bands at 2922 and 2364 cm^− 1^ are associated with the absorption bands of asymmetric CH and N-H, respectively. The peak appearing at 3448 cm^− 1^ signifies the existence of carboxylic groups, whereas the signal at 617 cm^− 1^ was accredited to disulfide S-S stretch^33,53^. The existence of these vibrations in alum sludge and conditioned sludge signify that it is possess cationic positively charged groups^54^

##### Various conditioners effectiveness on particle size and zeta potential of alum sludge

###### ζ-Potential

**Fig. 14:**
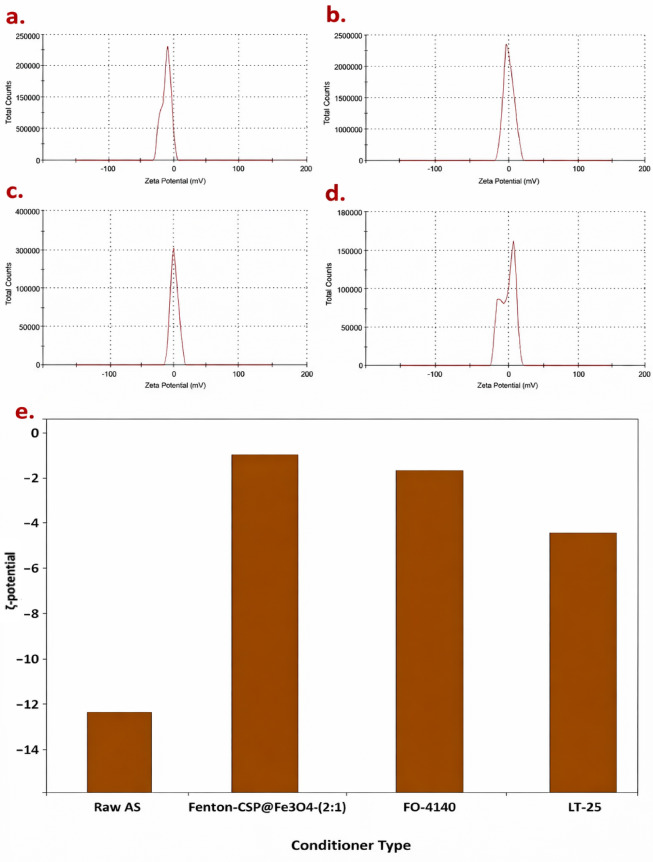
*ζpotential* shift of raw and conditioned alum sludge under different conditioners: (**a**) raw alum sludge, (**b**) Fenton conditioned sludge, (**c**) cationic polyelectrolyte FO-4140 conditioned sludge, (**d**) anionic polyelectrolyte LT-25 conditioned sludge and (**e**) *ζ-potential* comparison of different conditioners.

Zeta potential (*ζ-Potential)* is an essential indicator for sludge conditioning. *ζ-Potential* reveals the degree of repulsion between adjacent and similarly charged particles for alum sludge particles. Such measurement was developed for the validation of charge formation on alum sludge particles. The influence of conditioner type on ζ-potential is exhibited in Fig. 14. Generally, *ζ-Potential* influences the size and density of produced flocs. *ζ-Potential* value is associated with the repulsive force between alum sludge molecules particles and the space over the particles. Since each particle could repel each other, the coagulation process is prevented or difficult to occur. The extreme positive or negative charge values of *ζ-Potential* means that the alum sludge molecules are constant and also are difficult to coagulate as seen in the raw alum sludge in Figs. 14a and e. The reduction in *ζ-Potential* increases the flocculation tendency. On the other hand, the minimal positive or negative charge values of *ζ-Potential* exhibited the molecules are unstable and the coagulation process could be proceeded easily^55,56^. This is in agreement with the results of the conditioned alum sludge in Fig. 13b-d. Since electrostatic interactions are critical in the flocculation systems and hence measure of electrostatic potential is controlling the conditioning process. When the alum sludge associates other charged materials, higher magnitude of this *ζ-Potential*, the higher repulsion and/or attraction forces of alum sludge surface interactions^16,56^.

Initially, the *ζ-potential* of the raw alum sludge − 12.2 mV. The more negative *ζ-Potential*, the more turbid the supernatant since the suspended particles developed efficiently stabilized in the supernatant due to mutual repulsion. A good floc could be produced in a *ζ-Potential* ranged from − 8.0 mV to + 3.0 mV^16^. Adding positively charged coagulant might neutralize the negatively charged molecules and vice versa. Once the CSP@Fe_3_O_4_ conditioner was added, the *ζ-potential* of the alum sludge was significantly increased to reach to 0.263 mV when the sludge was conditioned. Across a wide pH range, CSP@Fe_3_O_4_ displayed a high positive surface charge and the isoelectric point exhibited at pH 9.0. Consequently, alum sludge with neutral potential exposes the attainment of an enhancement in alum sludge dewaterability performances. Thus, such lowering the *ζ-potential* of the conditioned sludge by Fenton’s reaction might be attributed to the presence of CSP@Fe_3_O_4_ that declining the ionization influence of the anionic charged of such functional groups at the operational conditions^57^. The potential of the alum sludge changed to nearly neutral, demonstrating that electrostatic repulsion was deduced between the sludge particles. Hence, sludge flocs can gather together easily and therefore the water in the sludge is released due to the extrusion force. Such examination is in agreement with the aforementioned cited studies stated in the previous published data^58^.

*ζ-potential* of the sludge was significantly improved when different conditioners were added. *ζ-potential* ranged from − 0.371 to -3.22 mV when the cationic polymer FO-4140 and anionic polymer LT-25 polyelectrolyte conditioners were applied, respectively. The trend of reduction in the zeta of the conditioned sludge compared to the raw alum sludge may be beneficial to better sludge dewaterability. The cationic 4141 polymer already neutralized a portion of negative surface charge on the sludge surface. As coagulant is added, the *ζ-potential* approaches zero. The *ζ-potential* of LT 25 was distantly altered to a value lesser than that of 4140. Hence, alum sludge can significantly differ in their dewatering performance according to the type of conditioner used and the positive charge presents in the surface molecules of cationic polymer or CSP@Fe_3_O_4_ exhibited better coagulation environment. The *ζ-potential* of FO-4140 was slightly altered to a value lesser than that of LT 25. *ζ-potential* reaches to about zero, which is a critical point when CSP@Fe_3_O_4_ conditioner is added that is associated with the isoelectric point. The charge neutralization capacity of the flocculants controlled with a conditioner diminished in the following order: CSP@Fe_3_O_4_, LT-25 polymer and FO-4140 polyelectrolyte. It is noteworthy to mention that in different ways, such broadening of the *ζ-potential* distribution is associated to the higher ionic strength that enhances the surface charges. The extra negative zeta potential of could be clarified by the existence of free carboxylic group that alters the surface characteristics of the alum sludge^59^. Thus, agglomeration of the particles is induced and the flocs become more compact.

##### Floc size, particle size distribution (PSD) and dewatering routine

**Fig. 15:**
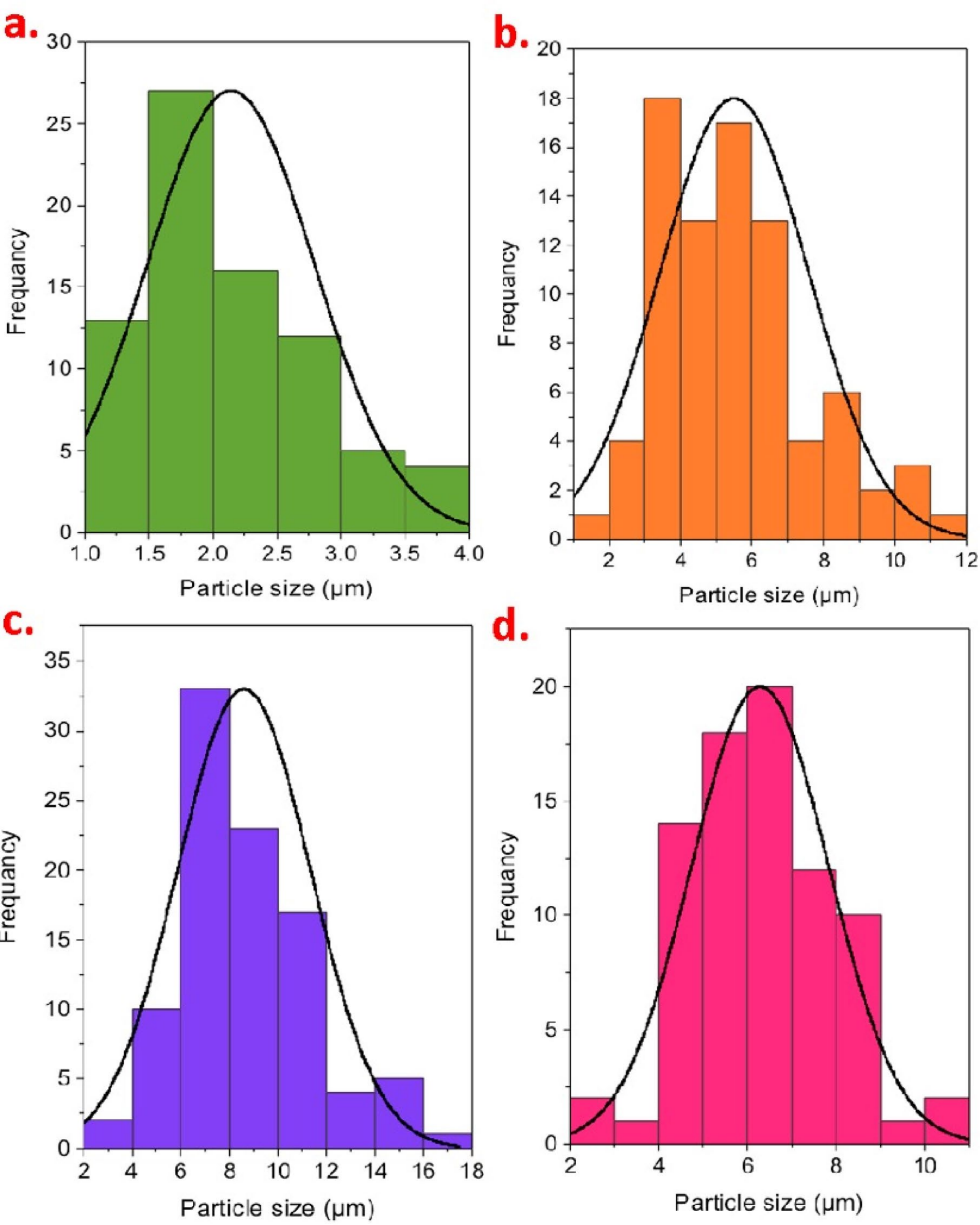
Floc size distribution before and after flocculation for alum sludge (**a**) raw AS and conditioned AS by (**b**) Fenton- CSP@Fe_3_O_4_ conditioner; (**c**) Magnafloc FO-4140 conditioner and (**d**) anionic Magnafloc LT-25 conditioner.

Compared to the raw alum sludge, lower segments of small particles for the conditioned samples that commonly had better dewatering performance (Fig. 15 (b, c and d). Demonstrated in Fig. 15, conditioned samples generally had better dewatering effectiveness, which seems to be due to minimum fractions of small particles and bigger particles of flocs compared to the raw alum sludge (Fig. 15a. Small flocs of the alum sludge particles exhibited poorer dewatering performance. The particle size analysis of sludge flocs treated with the CSP@Fe₃O₄ conditioner revealed a clear size distribution rather than a single uniform size. The minimum floc size was 2.87 μm, the maximum size reached 11.21 μm, and the average PSD was 5.65 μm. This range indicates effective aggregation induced by the CSP@Fe₃O₄ conditioner, with the formation of larger, denser flocs that enhance settling and dewatering performance. The presence of smaller flocs reflects some heterogeneity in aggregation, but overall, the shift toward larger floc sizes improves sludge compaction and water release. These observations confirm that the flocculation mechanism facilitated by CSP@Fe₃O₄ through a combination of iron ion bridging and chitosan-mediated polymer interactions that promotes floc growth and contributes to improved dewaterability, consistent with the trends observed in capillary suction time (CST) and sludge volume reduction.

On the other hand, the conditioned AS samples exhibited larger floc size as indicated by the data in Fig. 15b-d and therefore a good dewaterability is expected. Such observations could be consistent with the step of agglomeration of the particles and better compact and bulk particles will be attained whereas it is initially lesser than of that of the raw alum sludge. For instance, polymers, i.e. cationic Magnafloc FO-4140 and anionic Magnafloc LT-25 are holding aggregates together as displayed in Fig. 15c and d, respectively. The median PSD after the Magnafloc FO-4140 conditioner is 8.61 μm with a minimal 2.98 μm and maximal PSD of 2.98 μm. However, after the Magnafloc LT-25 conditioner, the minimum 2.87 μm and maximum 10.18 μm with average PSD of 6.29 μm. To add up, when applying Fenton conditioner based CSP@Fe_3_O_4_ conditioner, the maximum sludge particle size flocs reached to 11.21 μm and the average PSD is 5.65 μm. PSD after conditioning is higher than that of raw alum sludge which improves dewatering performance after conditioning since a connection between dewatering and flocs’ particle size distribution. Larger particles are detected for conditioned sludge with CSP@Fe_3_O_4_ conditioner and Magnafloc FO-4140 since the surface charge is related to the tendency of flocculation. Thus, these results are logical that is confirmed as in the abovementioned section for higher CST reduction for both CSP@Fe_3_O_4_ conditioner and Magnafloc FO-4140 rather than using Magnafloc LT-25 conditioner. These explanations are supported by data cited by Tony et al.^26^ in dewatering alum sludge using various conditioners. Thus, flocculation effect of iron ions and chitosan polymer drove the alum sludge flocs to become larger and helped to enhance its dewaterability performances with environmentally benign material.

##### Mechanism of alum sludge conditioning using CSP@Fe_2_O_4_

Although alum sludge contains a substantial proportion of inorganic matter, its dewaterability is mainly governed by organic components, particularly extracellular polymeric substances (EPS) and bound water. The CSP@Fe₃O₄-based advanced oxidation process preferentially targets these organic fractions. Hydroxyl radicals generated during the Fenton-like reaction oxidize EPS, weaken sludge floc structure, and promote the release of bound water, thereby enhancing sludge conditioning and dewatering^60–62^. This process is further reinforced by the synergistic effects of polymeric bridging and magnetic Fe₃O₄, which facilitate floc aggregation and structural modification. Thus, the conditioning mechanism involves the combined actions of polymer-assisted flocculation, heterogeneous Fenton-like oxidation, and magnetic-induced floc reorganization. Initially, the polycationic polysaccharide component of CSP@Fe₃O₄ promotes charge neutralization between negatively charged sludge particles and EPS, reducing electrostatic repulsion and enabling effective polymer bridging. This interaction results in the formation of larger, denser, and more compact flocs^25,31^.

Further, under acidic conditions, Fe₃O₄ acts as a heterogeneous catalyst, activating the oxidant to generate highly reactive hydroxyl radicals. These radicals selectively oxidize organic constituents of the sludge, including EPS, proteins, and polysaccharides responsible for strong water retention and poor dewaterability. The oxidative degradation of these organic matrices disrupts the three-dimensional floc network, weakening sludge cohesion and facilitating the release of bound and interstitial water. Hence, as oxidation progresses, the breakdown of organic structures increases sludge compressibility and enhances water release. Concurrently, magnetic Fe₃O₄ nanoparticles contribute to floc densification and restructuring through magnetic interactions, improving particle aggregation and promoting efficient solid–liquid separation. Notably, the inorganic fraction of alum sludge, such as sand and mineral particles, remains largely unaffected, confirming that the observed performance improvement is primarily driven by targeted modification of organic components^25^.

**Fig. 16:**
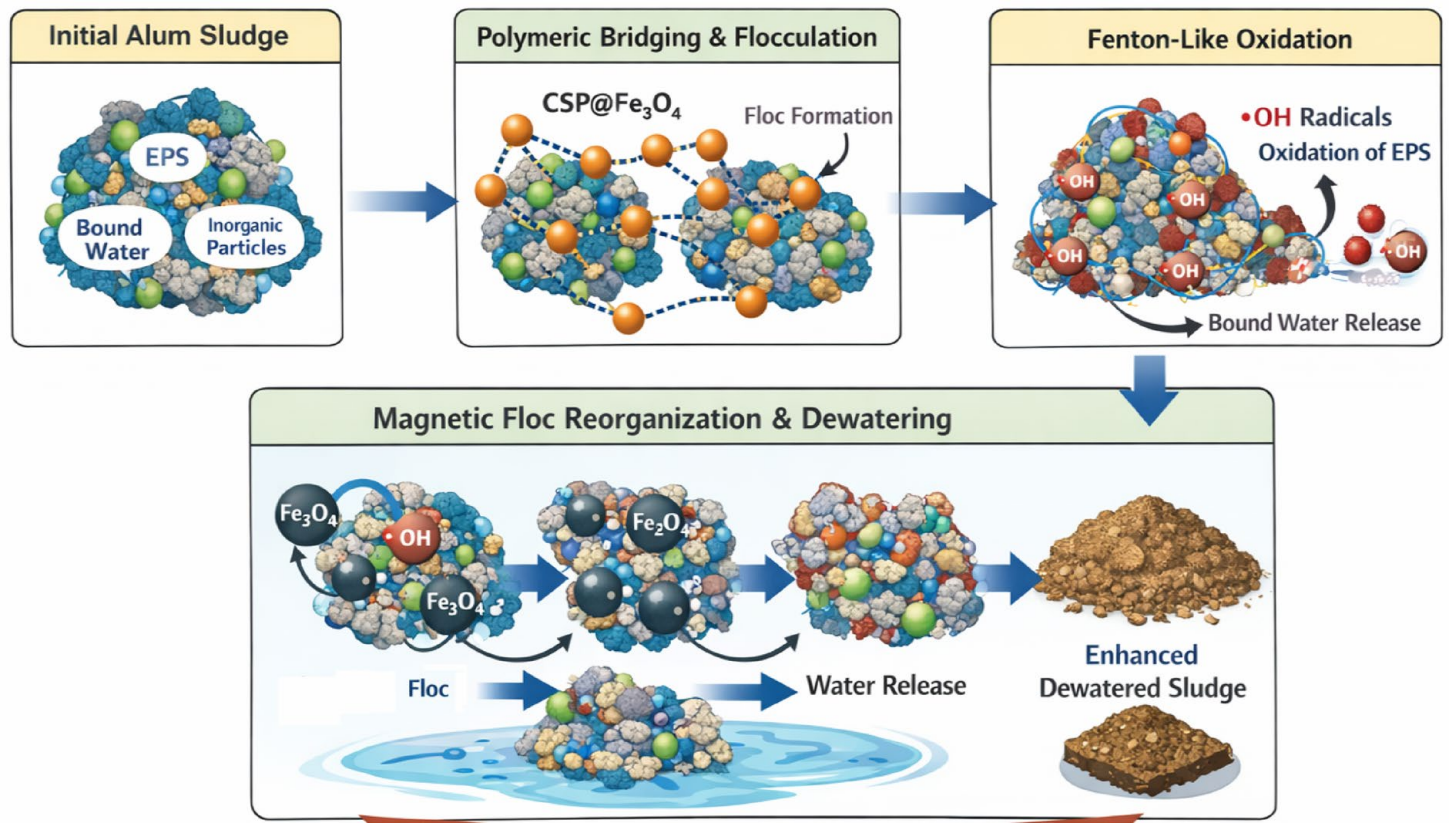
Mechanism of alum sludge conditioning using CSP@Fe₃O₄ nanocomposite.

Overall, the synergistic integration of polymeric bridging, radical-induced oxidation of EPS, and magnetic-assisted floc reorganization leads to significantly improved sludge conditioning and dewaterability. Additionally, the presence of catalytically active iron species offers potential for recovery and reuse of the conditioned sludge as a functional material in acidic Fenton-based wastewater treatment systems. The schematic illustration of such mechanism is graphically illustrated in Fig. 16.

Additionally, from the perspective of sludge treatment costs and environmental sustainability, CSP@Fe₃O₄ nanocomposites offer significant advantages over conventional commercial conditioning agents. Their dual functionality, combining polymeric flocculation with Fenton-like oxidation, enables higher sludge dewatering efficiency at lower material dosages, thereby reducing chemical consumption and minimizing secondary chemical residues in the treated sludge. The magnetic Fe₃O₄ component facilitates easy recovery and reuse of the composite over multiple cycles, which not only lowers operational costs but also reduces material waste and environmental release. Enhanced sludge dewaterability leads to a reduction in sludge volume and improves the efficiency of filtration or centrifugation processes, resulting in lower energy demand and decreased transportation and disposal requirements. Furthermore, the use of biodegradable, polysaccharide-based chitosan minimizes long-term environmental impacts and regulatory burdens compared with conventional inorganic or synthetic polymer conditioners, providing integrated economic, operational, and environmental benefits for sludge treatment applications.

##### Comparison of various sludge conditioning/dewatering by Fenton systems

Prior to various sludge discharge, it is essential to exposed to mechanical dewatering in that various facilities is applied such as press filters for the object of minimizing the water content^55^. Thus, suggesting reasonable chemical amenities conditioning system for higher sludge disposal is ever increasing by researchers. In this regard, Fenton’s reagent has been used as toxic less non-polyelectrolyte facility since it is an energy-saving technique as well as oxidizing recalcitrant substances in any type of sludge^60^. However, Fenton oxidation is a superior chemical oxidation dewatering facility, minimal research studies are available in comparison to the other chemical dewatering materials according to the cited literature^61^. Table 2 tabulated the scattered limited research work that is dealing with aluminum-based sludge discharged from drinking water treatment facilities as well as the other industrial wastewater sludge or municipal wastewater sludge by comparing their Fenton dewatering with the introduced current work.

According to the information displayed in Table 2, Fenton’s oxidation reaction system in its various forms is significantly effective and a reasonable conditioner for sludge dewatering that could replace commercial polyelectrolytes for chemical conditioning. However, the conditioning time are various from 1 min to 2 h according to the type of Fenton test as well as the kind of dewatered sludge. It is notably to remark that, the dewatering time in through currently proposed system is only 1.5 min in comparison to a longer time in other systems. Although the Fenton-Like conditioning system is only 1 min^62^, its dewaterability is only reached to 64% in comparison to 75% in the Fenton based CSP@Fe_3_O_4_-(2 −1) conditioner system presented in the current investigation. Also, it is significant to mention that CSP@Fe_3_O_4_-(2 − 1) based Fenton conditioner is dual treatment combining the polymer conditioning as an efficient conditioning system and an iron-based oxidation.

Generally, the effectiveness of chemical conditioners on enhancing alum sludge conditioning performance to improve its dewatering capability is controlled by various mechanisms. Although, using and applying solo conditioner has limited influences on sludge dewaterability. But, the composite conditioners might display pronounced presentation over a single sludge conditioner system. Also, the conditioned sludge particles by composite conditioners (dual conditioners) exhibited very condense layers of solid content. Moreover, chitosan is an environmentally friendly polyelectrolytes and supported a green environment in comparison to the other classical Fenton’s systems. Hence, this study introduced a green conditioning alternative option for dewatering water works sludge prior to mechanical dewatering.

**Table 2:** Comparison of various fenton’s conditioning systems for various sludge types.

Type of sludge	Sludge source and/or origin	Fenton’s conditioner type & operating conditions	Conditioning time	Sludge dewatering, %	Ref.
Alum sludge	Drinking water treatment plant, Menoufia, Egypt	Fenton based CSP@Fe_3_O_4_-(2 − 1) conditioner, Fe^2+^ 40 mg/L; H_2_O_2_ 400 mg/L; pH 3.0	1.5 min	75%	Current work
Citric acid waste water sludge	Secondary sedimentation tank from production of citric acid	Classical Fenton’s based FeSO_4_.7H_2_O, Fe^2+^ 80 mg/g-DS; H_2_O_2_ 20 mg/g-DS; pH 5.0	63 min	85.3% to 73.8%	^63^
Waste water sludge	Urban anaerobically digested sludge	Classical Fenton’s, Fe^2+^ 36 mM; H_2_O_2_ 360	60–120 min	98.8%	^64^
Waste water primary sludge	Wastewater Treatment plant, China	Classical Fenton’s, pH = 5.8, Fe^2+^ 0.1 g/g-TSS.	30 min	79%	^65^
Alum sludge	Sedimentation tank of Drinking Water Treatment Plant, Dublin, Ireland	Classical Fenton’s based FeCl_2_; Fe^2+^ 21 mg/g-DS + H_2_O_2_ 105 mg g-DS; pH 3.0	1.5 min	48%	^25^
Wastewater sludge	Wastewater treatment plant	Classical Fenton’s Fe^2+^ 6000 mg/L; H_2_O_2_ 5000 mg/L; pH 0.5–2.0.	60 min	76%	^66^
Municipal sewage sludge	Wastewater treatment plant, Wuxi City, China.	Classical Fenton’s Fe^2+^ 32 mg/g-DS; H_2_O_2_ 40 mg/g-DS; pH 3.0	60 min	69.95%	^34^
Alum sludge	Sedimentation tank of Drinking Water treatment Plant, Ballymore, Dublin, Ireland	Fenton-Like; Cu^2+^ 20 mg/g-DS + 125 H_2_O_2_mg/g-DS; pH 6.0	1 min	7%	^26^
Industrial Waste water sludge	Anaerobically digested sludge	Electro-Fenton, Fe^2+^ 15 mg/g-DS; H_2_O_2_ 25 mg/g DS	60 min	93.8%	^67^
Alum sludge	sedimentation tank of Drinking Water treatment Plant, Ballymore, Dublin, Ireland	Fenton-Like; Fe^3+^20 mg/g-DS; 125 H_2_O_2_mg/g-DS; pH 5.7	1 min	64%	^62^
Sewage sludge	Wastewater treatment plant, Guangzhou, China	Fenton/Lime; Fe^2+^ 50 mg/g-DS; H_2_O_2_ 30 mg/g DS; pH 3.0; lime 50 mg/g DS	90 min	96%	^32^
Municipal sewage sludge	secondary sedimentation tanks of five different municipal Wastewater Treatment Plants, Guangzhou, China	Fenton/Lime; Fe^2+^ of 50 mg/g DS, H_2_O_2_ of 30 mg/g DS; pH 3.0; lime 50 mg/g DS	120 min	95.13%	^68^
Alum sludge	sedimentation tank of Drinking Water treatment Plant, Kedwan, Minia city, Egypt	Solar-Fenton; Fe^3+^40 mg/g-DS; H_2_O_2_ 610 mg/g-DS; pH 7.0	180 min	94%	^62^
Alum sludge	Sedimentation tank of Drinking Water treatment Plant, Kedwan, Minia city, Egypt	Solar-Fenton; Fe^3+^50 mg/g-DS; H_2_O_2_ 800 mg/g-DS; pH 8.5	7 min	78%	^62^

*DS: Dry Solids .

## Conclusion

Various nanocomposite CSP@Fe_3_O_4_-(1–1), CSP@Fe_3_O_4_-(2 − 1) and CSP@Fe_3_O_4_-(1–3) based Fenton’s reagent conditioners were applied for improving alum sludge dewaterability regard. The optimal operational variables are optimized (CSP@Fe_3_O_4_ 40 mg/L and H_2_O_2_ of 400 mg/L at pH 3.0) for maximizing dewaterability which recorded 75% by using CSP@Fe_3_O_4_-(2 − 1) composite material. Further confirmation data demonstrated the change in particle size of the sludge through its morphology change after conditioning and the reduction in *ζ-Potential* increases the flocculation tendency. CSP@Fe_3_O_4_-(2 − 1) might deteriorate the electrostatic repulsion of alum sludge flocs and agglomerate minor molecules into larger particles which hence improving the dewatering performances. Also, the comparison with the commercial conditioners such as polyelectrolytes (LT-25 and FO-4140) and surfactant (SLS) was proposed and the results exhibited the importance of the current study in improving the sludge dewaterability with an environmentally friendly conditioner. All the above experimental results were verified by comparative data of sludge cake microstructure and microtopography after and prior to the addition of chemical conditioners. Also, chitosan/magnetite-based Fenton conditioner compared with the available Fenton’s conditioners in literature and the results demonstrated that CSP@Fe_3_O_4_ could be studied as an ideal alternative of available commercial conditioners since its unique superiority in dewaterability enhancement in an economic circular economy technique.

## Data Availability

The data that support the findings of this study are available from the corresponding author upon reasonable request.
